# A Novel Evaluation System of Psoriasis Curative Effect Based on Bayesian Maximum Entropy Weight Self-Learning and Extended Set Pair Analysis

**DOI:** 10.1155/2021/5544516

**Published:** 2021-04-17

**Authors:** Le Kuai, Xiao-ya Fei, Jing-si Jiang, Xin Li, Ying Zhang, Yi Ru, Ying Luo, Jian-kun Song, Wei Li, Shuang-yi Yin, Bin Li

**Affiliations:** ^1^Department of Dermatology, Yueyang Hospital of Integrated Traditional Chinese and Western Medicine, Shanghai University of Traditional Chinese Medicine, Shanghai, China; ^2^Institute of Dermatology, Shanghai Academy of Traditional Chinese Medicine, Shanghai, China; ^3^Center for Translational Medicine, Huaihe Hospital of Henan University, Kaifeng, Henan, China

## Abstract

**Background:**

Psoriasis is a complex skin disease and difficult to evaluate, and this study aimed to provide an objective and systematic approach for evaluating the efficacy of psoriasis.

**Methods:**

We sought to construct a Bayesian network from sixteen indicators in four aspects of psoriasis (skin lesion conditions, laboratory indexes, quality of life, and accompanying symptoms) and obtained weights of each index by combining the analytic hierarchy process with maximum entropy self-learning. Furthermore, we adopted stability analysis to calculate the minimum sample size of the system. The extended set pair analysis was utilized to evaluate the efficacy based on improved weights, which overcomes the limitation of set pair analysis (unable to evaluate the efficacy with uncertain grades and thresholds).

**Results:**

A total of 100 psoriasis vulgaris patients were included to evaluate the curative effect by the system. We obtained the weights of each index and the Euclidean distance for efficacy evaluation of 100 patients. The sensitivity analysis proved that the results had no significant change with the variation of single patient's indexes, which indicated that our results were stable to assess the effectiveness.

**Conclusions:**

We provided an available method of comprehensive effective evaluation of various indicators of psoriasis and based on both subjective and objective weights.

## 1. Introduction

Psoriasis is a complex, chronic, immune-mediated inflammatory skin disorder that affects approximately 125 million people worldwide [1]. Moreover, complications are associated with increased exacerbations in subjects with psoriasis, including diabetes, metabolic syndrome, and chronic obstructive pulmonary disease [2–4]. As a refractory systemic disease, psoriasis has a great impact on human health, and some can even be life threatening [5, 6]. Due to the complexity and severity, it is currently difficult to evaluate the efficacy of psoriasis [7]. Although varied evaluation tools and models have been practiced to evaluate the efficacy of diseases, there are still some shortcomings including the inability to integrate multiple indicators to construct an evaluation system and the lack of impartiality in evaluating the weights of various indicators [8–10].

In the Bayesian network, a directed acyclic graph is constructed to intuitively reflect the potential relationship between factors, and a conditional probability distribution table is used to reflect the strength of association [11]. A Bayesian network can reflect the multifactor relationship of psoriasis, so we adopted it to evaluate the efficacy. Meanwhile, the importance of each factor affecting the evaluation of the curative effect is dissimilar, so a reasonable weight needs to be provided [12]. The Analytic Hierarchy Process (AHP) is based on expert experience and was relatively prejudiced [13]. Interestingly, maximum entropy can identify the probability distribution that is most consistent with the cost function and makes the fewest assumptions [14]. To obtain the comparatively actual weight, we combined AHP with maximum entropy to maximize the entropy of the evaluation network through self-learning [15]. On the basis of the evidence mentioned above, we evaluated the efficacy quantitatively through extended set pair analysis (ESPA) based on multifactorial network and reasonable weights [16].

In the current study, we aimed to develop a comprehensive efficacy evaluation system of psoriasis vulgaris, based on Bayesian maximum entropy self-learning and ESPA. A total of 100 patients were included by this system for efficacy evaluation. It is expected to provide a new approach for curative effectiveness evaluation of psoriasis and other complex diseases.

## 2. Methods

### 2.1. Patients

Adult patients (between 18 and 65 years of age) were eligible to participate if they satisfied the condition of both Western and traditional Chinese medicine (TCM) diagnosis standards for psoriasis vulgaris. The trial was performed in accordance with our previous study [17]. The study was approved by the Ethics Committee of the Yueyang Hospital of Integrated Traditional Chinese and Western Medicine (approval no. 2019-028). All participants provided written informed consent before entering the study.

### 2.2. Case Study Design

Eligible patients received oral TCM herbal medication tailored to the participant's disease progression. Medication was administered twice every day during the intervention phase. After 8 weeks of treatments, clinical efficacy was assessed, and blood samples were collected for all eligible patients. The measurement of laboratory indexes was detected in accordance with our previous study [18], which is described in the supplementary materials.

### 2.3. Bayesian Network Construction

**Figure 1:**
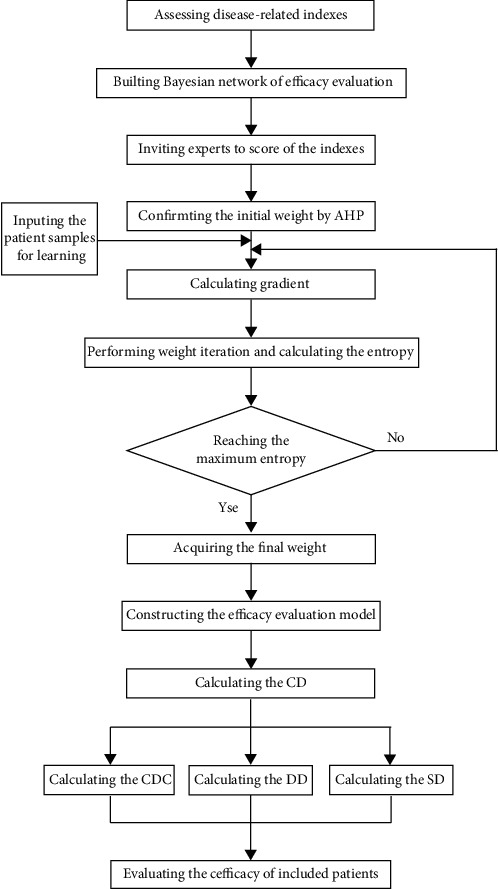
Schematic of the process.

A Bayesian network consists of a directed acyclic graph (DAG) and a series of conditional probability tables (CPTs). The nodes represent random variables, and edges represent the conditional dependences among nodes in a DAG [19]. In our study, the nodes were the indexes related to psoriasis. The conditional probability of each node was obtained according to the relationship between the indexes. An overview of the study flow is shown in Figure 1.

### 2.4. Calculation of the Initial Weight (IW) by the AHP

We invited experts to score according to the significance of the indexes (Supplementary Table S1). Then, the IWs of indexes were calculated by the AHP according to the following steps [20]. The scores were regarded by the expert, and the IWs were back in calculation if they failed to pass the consistency test.

### 2.5. Calculation of Maximum Entropy and the Final Weight (FW) by Self-Learning

In the process of self-learning, the maximum entropy was taken as the output condition by the gradient descent [21]. The IWs were input into the system to self-learn. Finally, the FW of the indexes was calculated according to the Bayesian network.

### 2.6. Efficacy Evaluation by ESPA

The efficacy of patients was evaluated by ESPA as described in the previous studies [16]. For the data of included patients in the index *k*, the greatest and smallest value of the data represented the upper threshold *u*_*k*_ and lower threshold *v*_*k*_, respectively. The assuming arbitrary value *x*_*kl*_ belongs to [*v*_*k*_, *u*_*k*_]. In this study, if the smaller values mean a better level of the effect, the connection degree (CD) of the patient *l* with respect to the index *k* could be calculated by equation (1). Conversely, if the greater values of data mean a lower level of efficacy, then the CD could be calculated by equation (2) [22].

(1) $$\begin{array}{c} \mu_{kl}=\frac{x_{kl}\left(x_{kl}-v_{k}\right)}{u_{k}\left(u_{k}-v_{k}\right)}+\frac{2\left(u_{k}-x_{kl}\right)\left(x_{kl}-v_{k}\right)}{u_{k}\left(u_{k}-v_{k}\right)}i+\frac{\left(u_{k}-x_{kl}\right)\left(u_{k}+v_{k}-x_{kl}\right)}{u_{k}\left(u_{k}-v_{k}\right)}j, \end{array}$$

(2) $$\begin{array}{c} \mu_{kl}=\frac{\left(u_{k}-x_{kl}\right)\left(u_{k}+v_{k}-x_{kl}\right)}{u_{k}\left(u_{k}-v_{k}\right)}+\frac{2\left(u_{k}-x_{kl}\right)\left(x_{kl}-v_{k}\right)}{u_{k}\left(u_{k}-v_{k}\right)}i+\frac{x_{kl}\left(x_{kl}-v_{k}\right)}{u_{k}\left(u_{k}-v_{k}\right)}j, \end{array}$$

where *μ*_*kl*_ denotes CD of the patient *l* with respect to the index *k*, *i* indicates the uncertainty coefficient of discrepancy and its value range is [−1,1], and *j* connotes the contradictory coefficient with the value defined as −1.

After that, the similarity degree (SD) was proposed to reflect a couple of patients' similarities, and the range of SD was [0,1]. Then, the ideal patient denoted a patient with the optimal response and was availed to make a comparison with the patients who are evaluated for the efficacy. As set pair analysis (SPA) defines [23], the CD of an ideal patient was calculated by the following equation:

(3) $$\begin{array}{c} u^{*}=1+0\cdot i+0\cdot j, \end{array}$$

where *u*^*∗*^ denotes the CD of an ideal patient.

Moreover, the Euclidean distance (ED) was set to evaluate the SD of each patients, and the specific calculation method was mentioned in Yan's study [16]. The comprehensive evaluation was confirmed, and the weights were also considered in the calculation of the ED [3]. The formula is given as follows:

(4) $$\begin{array}{c} d\left(\mu_{l},u^{*}\right)={\left\{\sum_{k=1}^{m}\left[\omega_{k}\left(1-S\left(\mu_{kl},u^{*}\right)\right)\right]\right\}}^{\left(1/2\right)}, \end{array}$$

where *d*(*μ*_*l*_, *u*^*∗*^) denotes the ED between the assessed patient *l* and the ideal patient, *μ*_*kl*_ connotes the CD of the assessed patient *l* in index *k*, *S*(*μ*_*kl*_, *u*^*∗*^) represents the SD of the CD for patient *l* and the ideal patient, and index *k* is the FW of index *k*.

### 2.7. Stability and Sensitivity Analysis

The stability analysis was practiced to test the rationality of the sample size. When the sample size changed, the corresponding entropy and self-learning times were determined by the Bayesian maximum entropy self-learning model. The sample size was set from 5 to 100, and the interval was 5. This process was repeated 20 times.

As inputs of some indexes are uncertain, the results were affected by the uncertainties. Consequently, a sensitivity analysis was performed to check the consistency. Each patient was set as the variable sample in turn, and data in each index were set as the variable inputs. Assuming an error of ±10% in the inputs determined, that is to say, the range of input values was between 90% and 110% of the reference values [24]. Herein, the interval of input values was set as 1%. Then, ED with different input values were made by ESPA.

## 3. Results

### 3.1. Results of the Index Weights

A total of 100 psoriasis vulgaris patients were included from outpatients of 20 clinical centers. The characteristics of the patients and 16 indicators are shown in Table 1. The details are provided in Supplementary Table S2.

**Figure 2:**
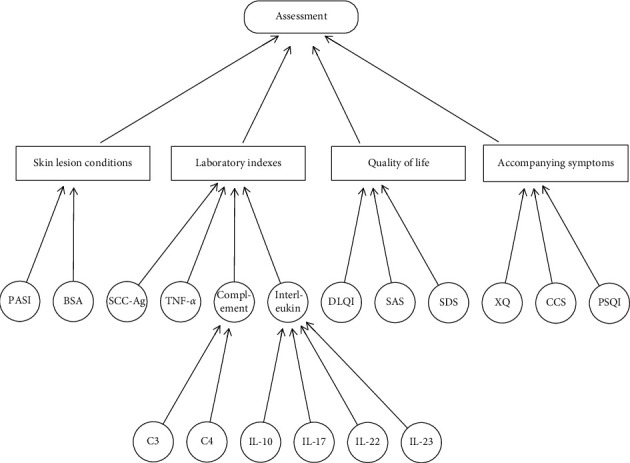
The Bayesian network of efficacy evaluation of psoriasis vulgaris.

To assess the efficacy of psoriasis vulgaris, we sought to construct the Bayesian network with four layers (Figure 2). The top layer was efficacy evaluation, that is, the target value of the network. The intermediate layers corresponded to the attribute values for the upper layer, and the target values for the next layer. The second layer consisted of four aspects of psoriasis vulgaris, including skin lesion conditions, laboratory indexes, quality of life, and accompanying symptoms. The third layer contained psoriasis area and severity index (PASI), body surface area (BSA) [25], squamous cell carcinoma antigen (SCC-Ag), tumor necrosis factor-*α* (TNF-*α*), interleukin (IL) and complement levels [26], Dermatology Life Quality Index (DLQI), Self-Rating Anxiety Scale (SAS) and Self-Rating Depression Scale (SDS) [27], the scales of Xerostomia Questionnaire (XQ), Cleveland Clinic Score (CCS), and Pittsburgh Sleep Quality Index (PSQI) [28–31]. Of these, IL comprised of IL-10, IL-17, IL-22, and IL-23, and the complement involved complement 3 (C3) and complement 4 (C4) [26]. Each node of the bottom layer was the attribute value of assessment, which was as well the outcome measure of this study.

Each abovementioned index contributed variously to the efficacy evaluation. Therefore, we confirmed the IW by the AHP according to expert score (Supplementary Table S3). Then, we performed consistency checks (Supplementary Table S4). The results passed the consistency check, so the weights were available for the efficacy evaluation, whereas the AHP required repeated artificial modification of the judgment matrix, and the evaluation of each index with different experts tended to be various [13]. Therefore, AHP was combined with the Bayesian network and self-learning to obtain more unbiased and accurate results. The Bayesian maximum entropy self-learning weights were obtained when the entropy values were the maximum. Furthermore, the FW was calculated according to the hierarchical relationships of the Bayesian network (Table 2).

To further demonstrate the availability of our method, we compared the weights from two methods (AHP and AHP with maximum entropy self-learning). The results revealed that the weight orderings of recognized indexes of psoriasis (PASI and BSA) were same, which confirmed our method was reliable to an extent. Interestingly, the weight orderings of complement (C3 and C4), quality of life (DLQI, SAS, and SDS), and accompanying symptoms (PSQI, XQ, and CCS) ascended after maximum entropy self-learning, which was consistent with the latest research that psoriasis is a systemic disease [1–4, 7]. Unexpectedly, the weights of IL-17, IL-22, and IL-23 were decreased that were correlated to the interaction between the interleukin family, or the specificity of interleukins in psoriasis is actually not high.

### 3.2. Stability Analysis

**Figure 3:**
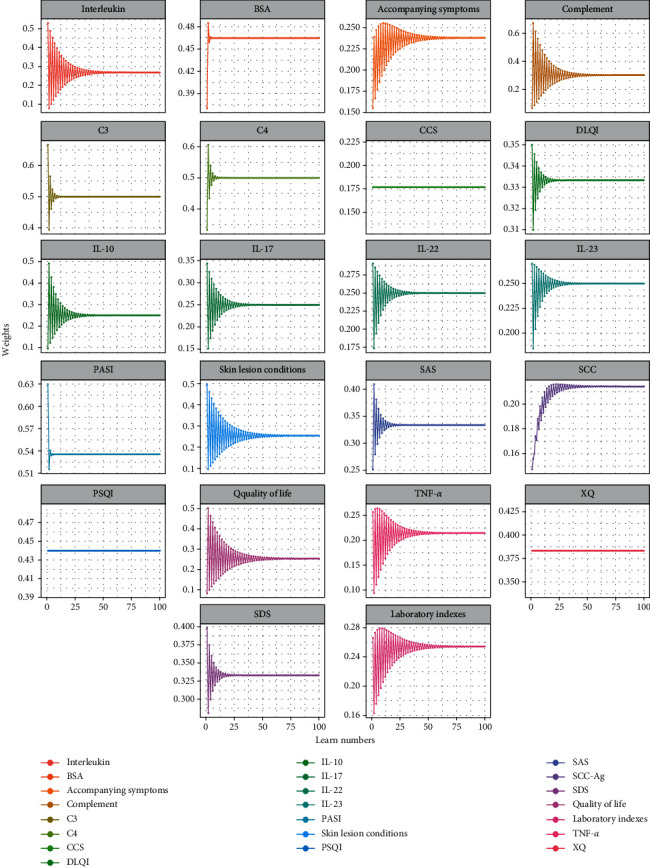
The weights of each index altered in self-learning progress.

**Figure 4:**
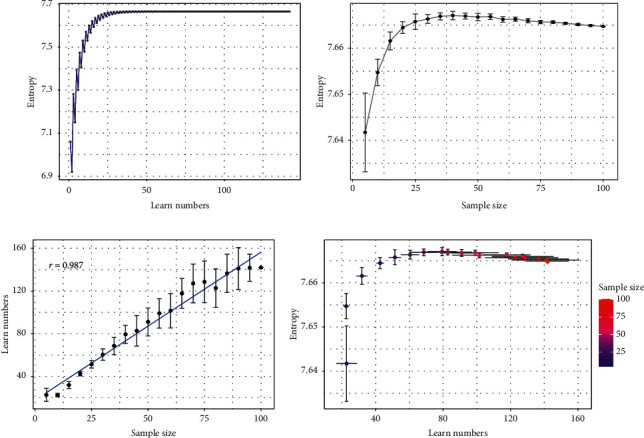
The stability and sensitivity analysis results of the evaluation system of the psoriasis curative effect. (a) The entropy was incremental and tended to be stable in increasing with numbers of self-learning when n is 100. (b) The maximum entropy varied when the sample size was from 5 to 100. (c) The self-learning times showed a linear increase (*r* = 0.987) with the growth of the sample size. (d) The relationship among entropy, self-learning times, and sample size.

**Figure 5:**
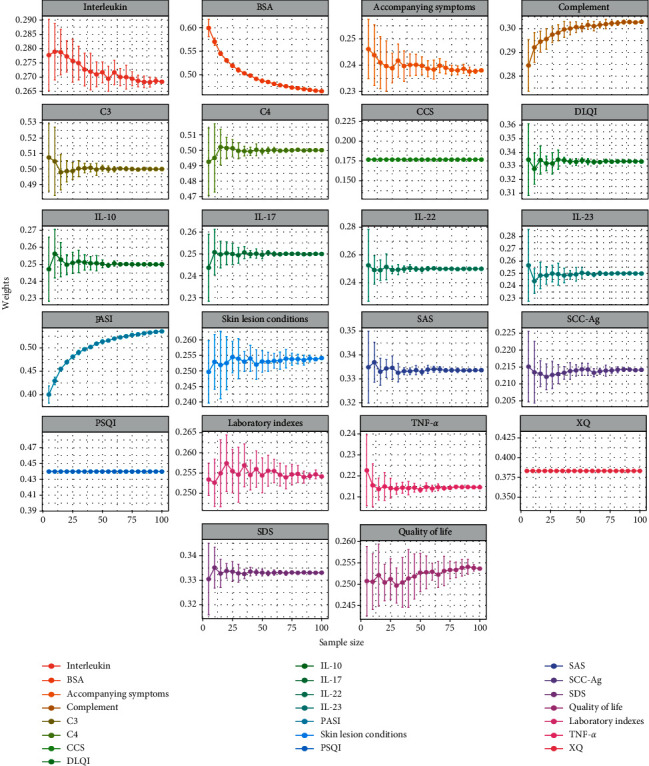
The weight altered when the sample size was from 5 to 100.

Considering the influence of sample size, stability analysis was carried out. The weights and entropy gradually altered and finally tended to be stable in the course of self-learning (Figures 3 and 4(a)). It indicated the results are relatively stable when the sample size is 100. On this basis, we explored the relationship among sample size, weights, self-learning times, and entropy values. When sample size input was increased, the entropy altered. In addition, the error bars of the entropy and weights were shorter gradually, indicating that the larger the sample size is, the more stable the entropy and weights are (Figures 4(b) and 5). Besides, the self-learning times showed a linear increase (*r* = 0.987) with the growth of the sample size (Figure 4(c)), revealing that the computing cost was enhanced with increasing sample size. Next, we showed the relations between the three. The vertical error bars and horizontal error bars varied with the numbers of self-learning increasing. With the increase of sample size, the entropy was more stable, and the numbers of self-learning increased, which reminded the three-way interaction (Figure 4(d)). Above, the stability analysis demonstrated that entropy increases from starting levels and becomes gradually stable with the growth of sample size and self-learning times. Approximately 50 patients were needed to make the model stable.

### 3.3. Results of Efficacy Evaluation

We included 100 patients for efficacy evaluation based on the results of stability analysis.

Table 3 shows means and standard deviations (Stds) of all patients with respect to each index (complete results are in the Supplementary Table S5). The SD reflected the similarities between the patient and the ideal patient. When the value of SD was closer to 1, it indicated that a single index of the patient being tested was more similar to that of an ideal patient.

Aggregating SD of each index to obtain ED: the ED from small to large corresponded to the efficacy from superior to inferior (Table 4). The results showed that the patients with preferable curative effect were L1 (0.3196), L18 (0.3713), L17 (0.3722), L37 (0.3906), and L21 (0.4126), whereas those with unfavorable effect were L100 (0.7904), L90 (0.7749), L61 (0.7656), L82 (0.7469), and L99 (0.7400). Interestingly, ESPA was feasible for efficacy evaluation, overcoming the major limitation of uncertain grades and thresholds.

### 3.4. Sensitivity Analysis

**Figure 6:**
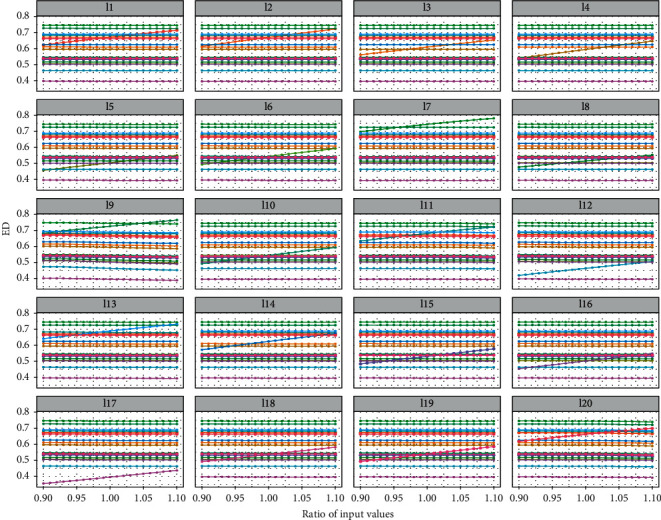
The other patients' ED changed when the input data of a patient were modified.

As the inputs of some indexes of efficacy evaluation are uncertain, efficacy evaluation results were affected by their uncertainties. Thus, a sensitivity analysis was performed to check the consistency of the obtained efficacy evaluation ranking. If the change of a patient's data affected the thresholds of the indexes, the ED of other patients changed accordingly. Sensitivity analysis was performed on 100 patients, where 20 patients are randomly shown in Figure 6. The other patients' ED had no noticeable changes when a patient's indicators were changed. This result illustrated that the ESPA efficacy evaluation model is relatively stable.

## 4. Discussion

Psoriasis is an immune-mediated chronic inflammatory skin disease with a high incidence. Till now, the question of how to comprehensively evaluate the treatment for psoriasis is a major clinical issue [32]. In this study, we first constructed a novel evaluation system of psoriasis by adopting Bayesian maximum entropy self-learning and ESPA.

The most obvious finding to emerge from the analysis was that PASI and BSA, as the most common evaluation tools of psoriasis, were the core indicators with the highest weights either before or after self-learning, which is consistent with expert knowledge and clinical experience. Guidelines of European Association of Dermatology and venereal Diseases recommended that PASI score and BSA should be the first choice of an objective indicator [33]. Even though the United States guidelines indicated that PASI score is cumbersome, it is still employed as a criterion for assessing the severity of psoriasis patients [34]. One previous study showed that compared with Patients Global Assessment, BSA and PASI have higher weights, which is consistent with our result [35].

One interesting finding was that the weights of C3 and C4 increased significantly in the self-learning system. Previous studies showed that the levels of C3 and C4 in patients with psoriasis were significantly higher, and C3 could be considered as a reliable marker of cardiac metabolic risk in psoriasis [36, 37]. Animal experiments have demonstrated that psoriasiform dermatitis was significantly alleviated and a marked reduction in C3 has been observed throughout when the S100A9 gene was deleted, in an imiquimod-induced mouse psoriasis model [38]. These studies indicate that there is a correlation between psoriatic lesions and C3 level, and the complement factor should be monitored as a considerable indicator of evaluation in various therapeutic interventions. Combining the AHP and maximum entropy criterion, we acquired more subjective and accurate results than the AHP on the weights of C3 and C4. However, C3 and C4 have not been integrated into the existing system, and the current study will fill this gap.

Besides, accompanying symptoms and quality of life received higher weights after self-learning. Previous research suggested that DLQI should be recognized as a major indicator and has even assisted in deciding patient-specific treatment strategies [27, 39]. In addition, the current study showed that the weight of SAS is higher than that of the AHP. One study has revealed that the psoriasis patients are more prone to anxiety than normal people, even though there is no obvious correlation between anxiety and the severity of psoriasis lesion, suggesting that doctors should not ignore the anxiety level of patients with mild psoriasis [40].

What is curious about this result is that the weight of interleukin was significantly lower than that by the AHP, and there were few differences among IL-10, IL-17, IL-22, and IL-23. In contrast, these indicators were considered as essential indicators in one of our earlier studies [26]. On the one hand, the reason may be related to the interactions among these indicators, and the evidence of the complex interplay has been proven. Previous studies reported that IL-23 stimulates the process of IL-17 secretion by Th17 cells, while the secretion of IL-22 needs to be encouraged by Th17 [41]. Moreover, enhancing IL-22 could lead to inhibit the production of IL-17 or stimulate the production of IL-10 [42], indicating that there may be a mechanism of interaction among IL-17, IL-23, and IL-22, which needs to be further unraveled. Another possible explanation for this is that it likely relates to specificity of interleukin. The occurrence and progression of psoriasis is the result of the combined force of a series of inflammatory cytokines and signaling pathways, including IL-6, IL-17, IL-23, IL-27, TNF-*α*, and INF-*γ*, but only IL-17 is the core mediator directly involved in the inflammation and disease progression of psoriasis. However, it has been proved that the IL-17 family has been found to play a causative role in tumor progression and autoimmune disease [43]. Therefore, this phenomenon may be correlated with the complex downstream signaling pathways resulting in less specificity.

In order to guarantee the accuracy of unsupervised self-learning, we verified the stability and sensitivity of the system. The large sample sizes increased statistical power that makes the results reliable. With the increase of the sample size, the entropy values were gradually stable, and the numbers of self-learning were also gradually increasing. Moreover, we conducted sensitivity analysis arbitrarily changing the clinical data of patients one by one and found that EPSA results are almost unaffected by the sample size, which indicated that our results were stable.

Compared with the AHP, the present system contains the following characteristic and advantages. The main strength of this study is that we applied a Bayesian network to construct a comprehensive measurement of treatment effectiveness among a variety of symptom indicators. Secondly, the judgment matrix needs to repeatedly modify in the weight's calculation process by the AHP, and the scores assessed by a variety of experts have considerable variation by subjective factors. Therefore, on the basis of the known weights and self-learning method, the optimal weights were output according to the maximum entropy criterion, which has higher objectivity [44]. In addition, SPA or the set pair cloud model all relied on hierarchical classification so that the symptom grades and thresholds were needed during calculation of the CD. In the study, ESPA was an improvement over the SPA, which is applicable at uncertain grades and thresholds.

Despite these promising results, limitations remain. Firstly, the current study only included patients with psoriasis vulgaris, so that one should be cautious when generalizing to other types of psoriasis and the indicators may need to be adjusted. Secondly, a larger sample for model testing may be needed before practical application. Thirdly, considering the large numbers of indicators for psoriasis, other indicators could be included to evaluate the efficacy of psoriasis.

## 5. Conclusions

Given diversified indicators of psoriasis, we proposed a system that considers multiple indicators and uses more objective weights to evaluate the efficacy, while overcoming the application limitations of SPA. In addition, 100 patients were included to confirm the stability and effectiveness of the system through stability analysis and sensitivity analysis. Theoretically, the system can be applied to other diseases, which requires further research for clarification.

## Figures and Tables

**Table 1:** The characteristics of the patients and the indexes.

Characteristics	*n* or means	Percentages or stds	Minimums	Maximums
Male	59	59%	—	—
Ages, yrs	45.04	10.65	21	65
PASI	7.91	2.96	1.30	17.60
BSA (%)	9.57	2.84	3.00	17.00
SCC-Ag (ng/mL)	20.94	28.78	1.17	143.69
TNF-*α* (pg/mL)	9.38	4.49	3.70	41.70
C3 (g/L)	0.91	0.20	1.71	0.48
C4 (g/L)	0.47	2.38	0.13	24.00
IL-23 (pg/mL)	912.43	210.94	509.99	1446.99
IL-22 (pg/mL)	5.73	3.96	2.62	27.93
IL-17 (pg/mL)	5.07	1.77	1.93	9.11
IL-10 (pg/mL)	4.77	0.86	2.90	7.90
DLQI	5.58	3.18	0	15.00
SAS	36.75	9.13	25.00	68.75
SDS	32.44	10.10	25.00	80.00
XQ	16.20	9.31	0	43.00
CCS	9.74	4.97	0	21.00
PSQI	9.28	5.67	0	21.00

Stds, standard deviations; PASI, psoriasis area and severity index; BSA, body surface area; SCC-Ag, squamous cell carcinoma antigen; TNF-*α*, tumor necrosis factor-*α*; C3, complement 3; C4, complement 4; IL, interleukin; DLQI, Dermatology Life Quality Index; SAS, Self-Rating Anxiety Scale; SDS, Self-Rating Depression Scale; XQ, Xerostomia Questionnaire; CCS, Cleveland Clinic Score; PSQI, Pittsburgh Sleep Quality Index.

**Table 2:** Comparison of the weight by the AHP and FW.

Indexes			Weights by AHP	FW
*Skin lesion conditions*	PASI		0.3126	0.1362
	BSA		0.1839	0.1182
	SCC-Ag		0.0392	0.0544
	TNF-*α*		0.0682	0.0545

*Laboratory indexes*	Complement	C3	0.0119	0.0385
		C4	0.0059	0.0385
	Interleukin	IL-10	0.0134	0.0171
		IL-17	0.0483	0.0171
		IL-22	0.0409	0.0171
		IL-23	0.0380	0.0171

*Quality of life*	DLQI		0.0290	0.0845
	SAS		0.0209	0.0846
	SDS		0.0330	0.0844

*Accompanying symptoms*	XQ		0.0593	0.0912
	CCS		0.0274	0.0421
	PSQI		0.0681	0.1047

AHP, analytic hierarchy process; FW, final weight.

**Table 3:** The characteristics of SD of each index.

Indexes	PASI	BSA	SCC	TNF	C3	C4	IL-23	IL-22	IL-17	IL-10	DLQI	SAS	SDS	XQ	CCS	PSQI
Means	0.5512	0.5085	0.8359	0.8104	0.6274	0.9859	0.5832	0.8511	0.5620	0.4328	0.5792	0.7254	0.8555	0.5761	0.3095	0.5377
Stds	0.1497	0.1503	0.1904	0.1106	0.1336	0.0742	0.1753	0.1517	0.1971	0.1258	0.1929	0.1770	0.1738	0.2003	0.1007	0.2085

SD, similarity degree.

**Table 4:** The ED of 100 patients.

Serial number	Patients	ED
1	L1	0.3196
2	L18	0.3713
3	L17	0.3722
4	L37	0.3906
5	L21	0.4126
6	L6	0.4328
7	L16	0.4449
8	L14	0.4507
9	L32	0.4563
10	L3	0.4591
11	L10	0.4791
12	L29	0.4813
13	L35	0.4843
14	L26	0.4851
15	L5	0.4872
16	L15	0.4939
17	L38	0.4966
18	L8	0.4974
19	L19	0.5016
20	L62	0.5059
21	L51	0.5070
22	L28	0.5123
23	L31	0.5132
24	L46	0.5152
25	L2	0.5168
26	L57	0.5237
27	L27	0.5249
28	L7	0.5250
29	L50	0.5287
30	L11	0.5290
31	L53	0.5293
32	L23	0.5318
33	L9	0.5348
34	L59	0.5354
35	L25	0.5363
36	L40	0.5411
37	L78	0.5411
38	L41	0.5423
39	L4	0.5452
40	L73	0.5468
41	L64	0.5581
42	L52	0.5611
43	L36	0.5616
44	L42	0.5642
45	L75	0.5765
46	L76	0.5837
47	L44	0.5852
48	L92	0.5905
49	L45	0.5915
50	L33	0.5918
51	L34	0.5921
52	L63	0.5951
53	L22	0.5971
54	L58	0.6017
55	L56	0.6055
56	L72	0.6070
57	L83	0.6080
58	L66	0.6082
59	L71	0.6091
60	L97	0.6136
61	L20	0.6148
62	L77	0.6192
63	L55	0.6212
64	L49	0.6218
65	L86	0.6225
66	L30	0.6231
67	L70	0.6242
68	L95	0.6297
69	L87	0.6385
70	L47	0.6454
71	L13	0.6520
72	L74	0.6609
73	L80	0.6618
74	L88	0.6664
75	L65	0.6691
76	L94	0.6696
77	L91	0.6725
78	L96	0.6753
79	L39	0.6772
80	L81	0.6785
81	L69	0.6801
82	L79	0.6807
83	L68	0.6823
84	L24	0.6829
85	L67	0.6846
86	L54	0.6895
87	L89	0.6953
88	L12	0.7016
89	L48	0.7036
90	L60	0.7108
91	L43	0.7213
92	L85	0.7264
93	L84	0.7330
94	L94	0.6696
95	L95	0.6297
96	L96	0.6753
97	L97	0.6136
98	L98	0.7360
99	L99	0.7400
100	L100	0.7904

ED, Euclidean distance.

## Data Availability

All of the data used to support the findings of this study are available from the corresponding author upon request.

## Abbreviations

AHP: Analytic hierarchy process
ESPA: Extended set pair analysis
TCM: Traditional Chinese medicine
DAG: Directed acyclic graph
CPTs: Probability tables
IW: Initial weight
FW: Final weight
CD: Connection degree
SD: Similarity degree
SPA: Set pair analysis
ED: Euclidean distance
PASI: Psoriasis area and severity index
BSA: Body surface area
SCC-Ag: Squamous cell carcinoma antigen
TNF-a: Tumor necrosis factor-*α*
IL: Interleukin
DLQI: Dermatology Life Quality Index
SAS: Self-Rating Anxiety Scale
SDS: Self-Rating Depression Scale
XQ: Xerostomia Questionnaire
CCS: Cleveland Clinic Score
PSQI: Pittsburgh Sleep Quality Index
C3/4: Complement 3/4
Stds: Standard deviations.

## Supplementary Materials

Supplementary Materials Supplementary Table S1: experts' judgment on importance of each index. Supplementary Table S2: the data of each index of 100 patients. Supplementary Table S3: expert scores of the indexes. Supplementary Table S4: results of the weights calculated by the AHP and the consistency test. Supplementary Table S5: the SD of 100 patients.

## References

[1] Armstrong A. W., Read C. (2020). Pathophysiology, clinical presentation, and treatment of psoriasis. *JAMA*.

[2] Armstrong A. W., Harskamp C. T., Armstrong E. J. (2013). Psoriasis and metabolic syndrome: a systematic review and meta-analysis of observational studies. *Journal of the American Academy of Dermatology*.

[3] Fang N., Jiang M., Fan Y. (2016). Association between psoriasis and subclinical atherosclerosis: a meta-analysis. *Medicine*.

[4] Li X., Kong L. J., Li F. L. (2015). Association between psoriasis and chronic obstructive pulmonary disease: a systematic review and meta-analysis. *PLoS One*.

[5] Simoes J. F., Ribeiro J., Ferreira B. R. (2015). The role of tonsillectomy in psoriasis treatment. *BMJ Case Reports*.

[6] Takeichi T., Akiyama M. (2020). Generalized pustular psoriasis: clinical management and update on autoinflammatory aspects. *American Journal of Clinical Dermatology*.

[7] Michalek I. M., Loring B., John S. M. (2016). *WHO Global Report on Psoriasis*.

[8] Brugnara G., Isensee F., Neuberger U. (2020). Automated volumetric assessment with artificial neural networks might enable a more accurate assessment of disease burden in patients with multiple sclerosis. *European Radiology*.

[9] Xu W., Zhao Y., Nian S. (2018). Differential analysis of disease risk assessment using binary logistic regression with different analysis strategies. *Journal of International Medical Research*.

[10] Codoñer F. M., Ramírez-Bosca A., Climent E. (2018). Gut microbial composition in patients with psoriasis. *Scienticfic Report*.

[11] Pan J., Ren Z., Li W. (2018). Prevalence of hyperlipidemia in Shanxi Province, China and application of Bayesian networks to analyse its related factors. *Scientific Report*.

[12] Liu J., Chen M., Yang T., Wu J. (2018). IoT hierarchical topology strategy and intelligentize evaluation system of diesel engine in complexity environment. *Sensors*.

[13] Zou Q., Zhou J., Zhou C., Song L., Guo J. (2013). Comprehensive flood risk assessment based on set pair analysis-variable fuzzy sets model and fuzzy AHP. *Stochastic Environmental Research and Risk Assessment*.

[14] Kimura Y. T., Takahashi D. Y., Holmes P. (2017). Vocal development in a Waddington landscape. *Elife*.

[15] Xu S., Cheng J., Quan Z. (2017). Reconstructing all-weather land surface temperature using the bayesian maximum entropy method over the Tibetan plateau and Heihe river basin. *IEEE Journal of Selected Topics in Applied Earth Observations and Remote Sensing*.

[16] Yan F., Xu K. L., Li D. S. (2017). A novel hazard assessment method for biomass gasification stations based on extended set pair analysis. *PLoS One*.

[17] Luo Y., Ru Y., Sun X. (2019). Characteristics of psoriasis vulgaris in China: a prospective cohort study protocol. *Annals of Translational Medicine*.

[18] Kuai L., Song J.-K., Zhang R.-X. (2020). Uncovering the mechanism of Jueyin granules in the treatment of psoriasis using network pharmacology. *Journal of Ethnopharmacology*.

[19] Bryois J., Buil A., Evans D. M. (2014). Cis and trans effects of human genomic variants on gene expression. *PLoS Genetics*.

[20] Jiang X., Xu S., Liu Y., Wang X. (2015). River ecosystem assessment and application in ecological restorations: a mathematical approach based on evaluating its structure and function. *Ecological Engineering*.

[21] Zhang H. B., Chen W. J., Yan M. (2018). The Bayesian maximum entropy weight self-learning method in the multimodal transport path optimization model. *Computer Applications and Software*.

[22] Yang F.-G., Liang Y., Singh V. P. (2014). Debris flow hazard assessment using set pair analysis models: take Beichuan county as an example. *Journal of Mountain Science*.

[23] Wang T., Chen J.-S., Wang T., Wang S. (2015). Entropy weight-set pair analysis based on tracer techniques for dam leakage investigation. *Natural Hazards*.

[24] Scarponi G. E., Guglielmi D., Casson Moreno V., Cozzani V. (2016). Assessment of inherently safer alternatives in biogas production and upgrading. *AICHE Journal*.

[25] Henseler T., Schmitt-Rau K. (2008). A comparison between BSA, PASI, PLASI and SAPASI as measures of disease severity and improvement by therapy in patients with psoriasis. *International Journal of Dermatology*.

[26] Li X., Xiao Q. Q., Li F. L. (2016). Immune signatures in patients with psoriasis vulgaris of blood-heat syndrome: a systematic review and meta-analysis. *Evidence-based Complementary and Alternative Medicine*.

[27] Hägg D., Sundström A., Eriksson M., Schmitt-Egenolf M. (2015). Decision for biological treatment in real life is more strongly associated with the psoriasis area and severity index (PASI) than with the dermatology life quality index (DLQI). *Journal of the European Academy of Dermatology and Venereology*.

[28] Feng L., Kan Z., Cao X. X. (2012). To establish the syndrome-typing model of psoriasis vulgaris blood-heat syndrome based on the set pair analysis method. *Chinese Journal of Integrated Traditional and Western Medicine*.

[29] Jiang N., Wei S., Mrtensson J. (2019). Assessment of radiation-induced xerostomia: validation of the xerostomia questionnaire in Chinese patients with head and neck cancer. *Cancer Nursing*.

[30] Heilmann C., Ruh B., Gall C. (2009). Preoperative prediction of survival for ventricular assist device (VAD) patients. *Thoracic & Cardiovascular Surgeon*.

[31] Pilz L. K., Keller L. K., Lenssen D. (2018). Time to rethink sleep quality: PSQI scores reflect sleep quality on workdays. *Sleep*.

[32] Fink C., Uhlmann L., Klose C. (2018). Automated, computer-guided PASI measurements by digital image analysis versus conventional physicians’ PASI calculations: study protocol for a comparative, single-centre, observational study. *BMJ Open*.

[33] Nast A., Spuls P. I., van der Kraaij G. (2017). European S3-guideline on the systemic treatment of psoriasis vulgaris-update apremilast and secukinumab-EDF in cooperation with EADV and IPC. *Journal of the European Academy of Dermatology and Venereology*.

[34] Singh J. A., Guyatt G., Ogdie A. (2019). Special article: (2018) American college of rheumatology/national psoriasis foundation guideline for the treatment of psoriatic arthritis. *Arthritis Care & Research*.

[35] Bożek A., Reich A. (2017). The reliability of three psoriasis assessment tools: psoriasis area and severity index, body surface area and physician global assessment. *Advances in Clinical and Experimental Medicine: Official Organ Wroclaw Medical University*.

[36] Öztürk G., Erbaş D., Gelir E., Gülekon A., İmir T. (2001). Natural killer cell activity, serum immunoglobulins, complement proteins, and zinc levels in patients with psoriasis vulgaris. *Immunological Investigations*.

[37] Torres T., Bettencourt N., Mendonça D., Vasconcelos C., Silva B. M., Selores M. (2014). Complement C3 as a marker of cardiometabolic risk in psoriasis. *Archives of Dermatological Research*.

[38] Schonthaler H. B., Guinea-Viniegra J., Wculek S. K. (2013). S100A8-S100A9 protein complex mediates psoriasis by regulating the expression of complement factor C3. *Immunity*.

[39] Liluashvili S., Kituashvili T. (2019). Dermatology life quality index and disease coping strategies in psoriasis patients. *Advances in Dermatology and Allergology*.

[40] Yu S., Tu H.-P., Huang Y.-C., Lan C.-C. E. (2019). The incidence of anxiety may not be correlated with severity of psoriasis: a prospective pilot study. *Medical Hypotheses*.

[41] Wu M., Deng Y., Li S. (2018). The immunoregulatory effects of traditional Chinese medicine on psoriasis via its action on interleukin: advances and considerations. *The American Journal of Chinese Medicine*.

[42] Kanda N., Kamata M., Tada Y., Ishikawa T., Sato S., Watanabe S. (2011). Human *β*-defensin-2 enhances IFN-*γ* and IL-10 production and suppresses IL-17 production in T cells. *Journal of Leukocyte Biology*.

[43] Tan J., Liu H., Huang M. H. (2020). Small molecules targeting ROR*γ*t inhibit autoimmune disease by suppressing Th17 cell differentiation. *Cell Death and Disease*.

[44] Kimball A. B., Gladman D., Gelfand J. M. (2008). National Psoriasis Foundation clinical consensus on psoriasis comorbidities and recommendations for screening. *Journal of the American Academy of Dermatology*.

