# Supplementary material for: A Novel Evaluation System of Psoriasis Curative Effect Based on Bayesian Maximum Entropy Weight Self-Learning and Extended Set Pair Analysis
Source: Evid Based Complement Alternat Med. 2021 Apr 17;2021:5544516. doi: 10.1155/2021/5544516 (PMC8075673; doi:10.1155/2021/5544516)
Supplement: Supplementary Materials — Supplementary Table S1: experts' judgment on importance of each index. Supplementary Table S2: the data of each index of 100 patients. Supplementary Table S3: expert scores of the indexes. Supplementary Table S4: results of the weights calculated by the AHP and the consistency test. Supplementary Table S5: the SD of 100 patients. [file 5544516.f1.docx]

**Supplementary**

# Supplementary methods

**Study design**

Eligible patients were randomly assigned (1:1:1) to blood heat syndrome group (BHS) group, blood stasis syndrome group (BSS) group and the non-blood heat nor blood stasis syndrome group (NHS) group. Participants will be grouped according to TCM syndrome classification and receive oral TCM herbal medication (according to the TCM syndrome classification, and tailored to the participant’s disease progression). Medication will be administered twice every day during the eight weeks of intervention. All patients were enrolled from the prospective cohort study [1]. Patients complicated with hepatic or renal functional disorders were not included in this trial.

A total of 100 patients of psoriasis vulgaris from Yueyang Hospital of Integrated Traditional Chinese and Western Medicine were included in this study. There were 26 males and 10 females in the BHS group, with an average age of (41.11 ±10.91) years, aged 21 to 65 years. There were 14 males and 21 females in the BSS group, aged from 29 to 64 years, with an average of (46.57 ±9.22) years. There were 19 males and 10 females in the NHS group, aged from 28 to 65 years old, with an average of (48.07 ±10.28) years. There was no significant difference in general data among the three groups (P > 0.05). During the treatment, 1 patient in the NHS group presented with stomach discomfort, and 1 in the BHS group showed nausea. There was 1 case of diarrhea in the BHS group and the NHS group, respectively. These symptoms were all mild and transient without recurrence after continuation of the treatment. No patients were withdrawn from the trial. Besides, blood routine, urine routine, hepatic and renal functions showed no significant changes before and after treatment.

**Assessments**

After 8 weeks treatment, all indicators was assessed and blood samples were collected. The levels of the laboratory indexes were measured using the SCC-Ag Human Enzyme-linked Immunosorbent Assay (ELISA) kit (DEIA6229, Creative-Diagnostics), TNF-α ELISA kit (ab181421, Abcam), IL-10 ELISA kit (ab185986, Abcam), IL-17 ELISA kit (ab119535, Abcam), IL-22 ELISA kit (ab216170, Abcam), IL-23 ELISA kit (ab221837, Abcam), C3 ELISA kit (ab108823, Abcam) and C4 ELISA kit (ab108824, Abcam). All procedures were performed according to the manufacturer’s instructions. The steps of the determination of concentration were documented in our previous study [2].

**Statistical analysis**

Analytical methods will be combined with descriptive analysis and comparative analysis. Qualitative data will be described by percentage, and a chi-square test will conduct comparative analyses. Quantitative data will be described as mean, standard deviation, minimum, and maximum. The t-test will be used for comparative analysis of data with normal distributions. Quantitative data exhibiting a non-normal distribution on the Wilcoxon rank-sum test will be analyzed using the Wilcoxon signed-rank sum test and covariance generalized linear models. The hypothesis test will be two-sided, and the test statistics and their corresponding p-values will be reported. A value of P<0.05 will be considered statistically significant. Collected data were analyzed using R software (version 3.6.0) by Shuang-yi Yin.

**References**

[1] Luo Y, Ru Y, Sun XY, et al. Characteristics of psoriasis vulgaris in China: a prospective cohort study protocol. Ann Transl Med. 2019;7:694.

[2] Kuai L, Song JK, Zhang RX, et al. Uncovering the mechanism of Jueyin granules in the treatment of psoriasis using network pharmacology. J Ethnopharmacol. 2020;262:113214.

# Supplementary Table S1 Experts judgment on importance of different indexes

| Linguistic Variables Levels | Value Range |
| --- | --- |
| Very important | (8, 10] |
| Important | (6, 8] |
| Semi-important | (4, 6] |
| Unimportant | (2, 4] |
| Very unimportant | (0, 2] |

# Supplementary Table S2 The data of each index of 100 patients

| Patients | Gender^a^ | Ages, yrs | PASI | BSA, % | SCC-Ag, ng/mL | TNF-α, pg/mL | C3, g/L | C4, g/L | IL-23, pg/mL | IL-22, pg/mL | IL-17, pg/mL | IL-10, pg/mL | DLQI | SAS | SDS | XQ | CCS | PSQI |
| --- | --- | --- | --- | --- | --- | --- | --- | --- | --- | --- | --- | --- | --- | --- | --- | --- | --- | --- |
| L1 | 1 | 36 | 12.1 | 13 | 45.61 | 10.30 | 1.10 | 0.30 | 1269.01 | 9.41 | 2.33 | 3.90 | 5 | 31.25 | 25.00 | 26 | 4 | 2 |
| L2 | 2 | 43 | 10.9 | 9 | 13.10 | 8.10 | 1.01 | 0.29 | 926.01 | 4.71 | 2.93 | 5.00 | 7 | 42.50 | 36.25 | 17 | 7 | 16 |
| L3 | 1 | 54 | 6.6 | 6 | 5.78 | 9.10 | 0.94 | 0.23 | 752.01 | 2.68 | 5.16 | 7.90 | 5 | 42.50 | 36.25 | 34 | 0 | 17 |
| L4 | 1 | 48 | 8.6 | 9 | 5.02 | 7.30 | 1.02 | 0.31 | 971.01 | 3.14 | 3.00 | 5.00 | 9 | 35.00 | 28.75 | 14 | 9 | 9 |
| L5 | 2 | 52 | 6.6 | 6 | 2.46 | 20.90 | 0.97 | 0.21 | 734.99 | 16.82 | 2.20 | 5.70 | 9 | 31.25 | 25.00 | 12 | 5 | 0 |
| L6 | 1 | 52 | 9.1 | 9 | 3.88 | 8.90 | 0.93 | 0.29 | 1116.00 | 5.29 | 4.01 | 3.10 | 1 | 31.25 | 25.00 | 0 | 13 | 8 |
| L7 | 1 | 28 | 16 | 14 | 56.47 | 12.30 | 0.87 | 0.16 | 1100.01 | 16.70 | 3.33 | 4.50 | 4 | 37.50 | 31.25 | 17 | 10 | 16 |
| L8 | 2 | 52 | 6.5 | 7 | 5.57 | 7.50 | 0.95 | 0.57 | 597.99 | 6.63 | 2.01 | 5.00 | 4 | 28.75 | 28.75 | 0 | 2 | 18 |
| L9 | 1 | 37 | 10.4 | 10 | 13.98 | 9.30 | 1.71 | 0.43 | 1204.99 | 3.14 | 5.66 | 5.00 | 5 | 36.25 | 30.00 | 23 | 12 | 4 |
| L10 | 2 | 61 | 9.4 | 7 | 2.63 | 8.90 | 0.78 | 0.19 | 1189.99 | 3.61 | 7.50 | 4.50 | 5 | 45.00 | 38.75 | 0 | 12 |  |
| L11 | 1 | 34 | 8.9 | 16 | 100.56 | 10.30 | 0.76 | 0.19 | 1212.00 | 4.33 | 2.48 | 5.00 | 9 | 41.25 | 42.50 | 29 | 15 | 3 |
| L12 | 1 | 62 | 6.5 | 6 | 1.21 | 8.10 | 1.08 | 0.17 | 722.01 | 3.49 | 3.58 | 5.00 | 3 | 25.00 | 25.00 | 14 | 12 | 14 |
| L13 | 2 | 34 | 8 | 11 | 6.95 | 6.70 | 0.90 | 0.23 | 939.00 | 4.14 | 1.93 | 4.40 | 10 | 58.75 | 80.00 | 0 | 10 | 11 |
| L14 | 2 | 41 | 10.8 | 12 | 24.14 | 6.30 | 0.92 | 0.26 | 1028.00 | 3.96 | 3.50 | 4.50 | 5 | 31.25 | 25.00 | 15 | 10 | 8 |
| L15 | 2 | 34 | 7.7 | 10 | 4.18 | 9.90 | 0.80 | 0.26 | 867.00 | 7.75 | 2.21 | 5.00 | 2 | 31.25 | 25.00 | 17 | 17 | 5 |
| L16 | 1 | 48 | 4.9 | 6 | 1.89 | 7.90 | 1.12 | 0.32 | 680.01 | 3.77 | 5.16 | 4.50 | 6 | 31.25 | 25.00 | 16 | 10 | 7 |
| L17 | 2 | 30 | 5 | 6 | 5.14 | 7.00 | 0.85 | 0.18 | 693.01 | 3.14 | 3.40 | 6.40 | 0 | 25.00 | 31.25 | 8 | 4 | 1 |
| L18 | 1 | 62 | 7.9 | 12 | 38.70 | 8.00 | 0.81 | 0.24 | 912.99 | 6.75 | 3.33 | 4.60 | 7 | 31.25 | 25.00 | 8 | 9 | 13 |
| L19 | 1 | 47 | 5.9 | 9 | 19.79 | 7.00 | 0.86 | 0.22 | 755.00 | 3.57 | 3.00 | 5.00 | 5 | 31.25 | 25.00 | 13 | 10 | 3 |
| L20 | 1 | 52 | 10 | 16 | 112.07 | 12.50 | 0.99 | 0.25 | 1127.01 | 7.12 | 6.16 | 4.50 | 2 | 28.75 | 25.00 | 23 | 9 | 18 |
| L21 | 2 | 39 | 8 | 10 | 17.09 | 8.90 | 0.96 | 0.21 | 945.00 | 5.98 | 3.81 | 3.50 | 6 | 37.50 | 31.25 | 7 | 3 | 5 |
| L22 | 1 | 53 | 8.4 | 9 | 4.94 | 4.30 | 0.60 | 0.20 | 953.01 | 4.24 | 4.44 | 4.50 | 7 | 32.50 | 26.25 | 9 | 13 | 10 |
| L23 | 2 | 63 | 8.1 | 10 | 28.02 | 6.90 | 0.99 | 0.18 | 955.99 | 3.82 | 3.10 | 6.20 | 4 | 35.00 | 28.75 | 28 | 8 | 6 |
| L24 | 1 | 37 | 8 | 12 | 27.37 | 7.40 | 0.86 | 0.20 | 927.00 | 4.33 | 3.75 | 4.50 | 5 | 40.00 | 33.75 | 26 | 13 | 14 |
| L25 | 1 | 60 | 10 | 12 | 36.73 | 12.10 | 0.85 | 0.16 | 1213.99 | 5.69 | 3.42 | 4.50 | 6 | 37.50 | 31.25 | 22 | 10 | 9 |
| L26 | 2 | 47 | 9.2 | 9 | 19.07 | 8.30 | 0.83 | 0.22 | 986.01 | 7.07 | 4.16 | 3.50 | 4 | 36.25 | 30.00 | 16 | 4 | 2 |
| L27 | 1 | 65 | 4.2 | 10 | 3.59 | 6.70 | 0.75 | 0.20 | 773.01 | 4.57 | 3.46 | 5.30 | 3 | 31.25 | 25.00 | 17 | 15 | 3 |
| L28 | 1 | 40 | 8.9 | 13 | 39.55 | 15.80 | 0.85 | 0.18 | 1127.01 | 7.28 | 4.50 | 4.90 | 8 | 48.75 | 42.50 | 10 | 8 | 7 |
| L29 | 1 | 50 | 13.8 | 11 | 28.03 | 10.50 | 1.16 | 0.37 | 1145.01 | 6.99 | 3.18 | 3.90 | 5 | 35.00 | 28.75 | 15 | 10 | 8 |
| L30 | 1 | 55 | 7.8 | 10 | 17.20 | 9.20 | 0.56 | 0.17 | 892.00 | 3.29 | 4.95 | 5.00 | 4 | 26.25 | 25.00 | 10 | 12 | 8 |
| L31 | 2 | 53 | 6.8 | 10 | 4.25 | 7.40 | 0.84 | 0.20 | 793.01 | 3.14 | 3.53 | 5.00 | 0 | 25.00 | 25.00 | 13 | 10 | 6 |
| L32 | 2 | 32 | 12 | 11 | 66.20 | 9.70 | 0.91 | 24.00 | 1065.99 | 15.45 | 3.78 | 5.00 | 6 | 37.50 | 31.25 | 33 | 6 | 21 |
| L33 | 2 | 63 | 2.6 | 5 | 6.91 | 6.50 | 1.04 | 0.25 | 612.01 | 3.51 | 4.16 | 5.40 | 6 | 35.00 | 37.50 | 19 | 10 | 10 |
| L34 | 1 | 21 | 4.3 | 11 | 23.69 | 9.50 | 0.75 | 0.13 | 681.00 | 3.78 | 4.61 | 4.80 | 4 | 36.25 | 30.00 | 9 | 3 | 3 |
| L35 | 2 | 62 | 6.4 | 7 | 3.94 | 9.20 | 0.73 | 0.30 | 723.00 | 5.73 | 3.50 | 4.50 | 9 | 33.75 | 27.50 | 19 | 21 | 2 |
| L36 | 2 | 29 | 6.7 | 3 | 1.17 | 6.10 | 0.91 | 0.24 | 749.00 | 3.14 | 5.00 | 4.90 | 2 | 28.75 | 25.00 | 6 | 10 | 16 |
| L37 | 1 | 43 | 9.7 | 9 | 7.91 | 4.00 | 1.59 | 0.21 | 848.01 | 3.30 | 2.64 | 5.30 | 0 | 32.50 | 26.25 | 24 | 6 | 4 |
| L38 | 1 | 38 | 4.7 | 9 | 11.40 | 9.80 | 1.30 | 0.35 | 596.01 | 3.14 | 3.58 | 4.50 | 5 | 30.00 | 25.00 | 16 | 13 | 6 |
| L39 | 2 | 37 | 5.4 | 10 | 17.42 | 6.40 | 0.79 | 0.13 | 705.00 | 3.14 | 4.33 | 5.00 | 2 | 28.75 | 25.00 | 27 | 8 | 21 |
| L40 | 1 | 40 | 10.4 | 8 | 5.96 | 4.30 | 0.79 | 0.20 | 975.99 | 3.14 | 3.66 | 3.30 | 4 | 40.00 | 33.75 | 0 | 14 | 7 |
| L41 | 1 | 57 | 4.6 | 8 | 6.38 | 7.40 | 0.83 | 0.20 | 695.01 | 5.68 | 5.16 | 5.00 | 9 | 43.75 | 37.50 | 19 | 19 | 15 |
| L42 | 2 | 54 | 10.3 | 15 | 65.83 | 9.50 | 0.50 | 0.26 | 1148.01 | 14.88 | 4.45 | 5.00 | 10 | 55.00 | 48.75 | 19 | 21 | 15 |
| L43 | 1 | 47 | 4.8 | 8 | 5.81 | 5.40 | 0.82 | 0.23 | 657.99 | 3.14 | 8.16 | 4.50 | 11 | 50.00 | 53.75 | 29 | 2 | 4 |
| L44 | 1 | 49 | 7.4 | 12 | 43.15 | 41.70 | 0.90 | 0.17 | 944.01 | 6.83 | 4.11 | 6.10 | 0 | 31.25 | 25.00 | 35 | 8 | 5 |
| L45 | 2 | 61 | 1.3 | 3 | 1.89 | 7.50 | 0.77 | 0.20 | 509.99 | 3.96 | 3.41 | 4.50 | 5 | 32.50 | 26.25 | 0 | 5 | 15 |
| L46 | 1 | 55 | 9 | 10 | 16.22 | 7.00 | 0.80 | 0.18 | 1110.99 | 3.90 | 3.60 | 5.00 | 6 | 33.75 | 27.50 | 12 | 9 | 3 |
| L47 | 1 | 46 | 8.6 | 8 | 6.75 | 3.70 | 0.98 | 0.24 | 993.01 | 3.40 | 3.50 | 5.00 | 5 | 32.50 | 26.25 | 31 | 6 | 20 |
| L48 | 1 | 54 | 2.9 | 5 | 5.37 | 8.20 | 0.90 | 0.20 | 687.01 | 5.21 | 4.83 | 5.00 | 2 | 26.25 | 25.00 | 13 | 14 | 16 |
| L49 | 2 | 38 | 1.9 | 7 | 9.22 | 5.50 | 1.06 | 0.29 | 611.01 | 3.54 | 5.15 | 4.50 | 6 | 31.25 | 25.00 | 3 | 8 | 18 |
| L50 | 1 | 44 | 11 | 11 | 21.88 | 11.90 | 0.82 | 0.23 | 1116.00 | 5.99 | 4.00 | 4.50 | 5 | 42.50 | 36.25 | 23 | 13 | 10 |
| L51 | 2 | 64 | 6.7 | 7 | 18.38 | 7.00 | 0.77 | 0.17 | 910.01 | 5.95 | 5.61 | 4.50 | 5 | 38.75 | 32.50 | 26 | 11 | 13 |
| L52 | 2 | 31 | 15.2 | 14 | 51.82 | 18.70 | 0.88 | 0.23 | 1397.01 | 27.93 | 3.83 | 4.50 | 6 | 36.25 | 30.00 | 10 | 1 | 7 |
| L53 | 1 | 35 | 6.3 | 8 | 4.76 | 17.00 | 1.06 | 0.25 | 741.99 | 5.45 | 5.73 | 4.50 | 2 | 25.00 | 25.00 | 15 | 12 | 14 |
| L54 | 1 | 39 | 8.6 | 11 | 22.04 | 10.80 | 0.99 | 0.20 | 1029.99 | 5.42 | 5.51 | 4.50 | 7 | 43.75 | 37.50 | 14 | 10 | 4 |
| L55 | 2 | 55 | 7 | 9 | 11.52 | 8.30 | 0.79 | 0.15 | 684.01 | 4.33 | 6.58 | 6.90 | 8 | 36.25 | 30.00 | 31 | 2 | 19 |
| L56 | 2 | 54 | 5.8 | 9 | 11.64 | 9.70 | 1.09 | 0.31 | 705.99 | 5.15 | 4.55 | 5.00 | 1 | 25.00 | 25.00 | 22 | 11 | 7 |
| L57 | 2 | 43 | 8 | 7 | 3.11 | 9.40 | 0.97 | 0.29 | 899.01 | 5.32 | 6.16 | 6.10 | 6 | 31.25 | 25.00 | 11 | 8 | 0 |
| L58 | 2 | 46 | 7.2 | 9 | 9.30 | 9.40 | 1.10 | 0.23 | 763.99 | 4.12 | 5.56 | 4.90 | 4 | 38.75 | 32.50 | 26 | 12 | 5 |
| L59 | 1 | 42 | 4.6 | 8 | 3.81 | 7.20 | 0.73 | 0.76 | 867.00 | 3.78 | 6.76 | 5.00 | 5 | 50.00 | 43.75 | 0 | 10 | 4 |
| L60 | 2 | 60 | 8.2 | 8 | 4.20 | 4.70 | 0.84 | 0.20 | 935.00 | 4.78 | 5.86 | 4.50 | 4 | 38.75 | 32.50 | 20 | 7 | 3 |
| L61 | 1 | 60 | 7.9 | 9 | 3.33 | 8.40 | 1.17 | 0.28 | 846.00 | 11.06 | 6.98 | 3.50 | 0 | 25.00 | 25.00 | 15 | 6 | 2 |
| L62 | 1 | 34 | 12.1 | 12 | 30.35 | 8.10 | 0.87 | 0.20 | 1149.00 | 12.94 | 5.70 | 4.80 | 0 | 30.00 | 25.00 | 9 | 6 | 18 |
| L63 | 2 | 46 | 7.4 | 9 | 9.24 | 13.90 | 0.92 | 0.24 | 868.99 | 5.84 | 4.00 | 4.20 | 9 | 42.50 | 36.25 | 16 | 18 | 3 |
| L64 | 1 | 42 | 13 | 9 | 6.24 | 3.90 | 1.06 | 0.17 | 1056.99 | 4.96 | 5.86 | 3.50 | 6 | 60.00 | 53.75 | 8 | 7 | 11 |
| L65 | 1 | 57 | 8.9 | 10 | 38.10 | 11.00 | 1.00 | 0.30 | 1204.99 | 5.15 | 2.03 | 4.50 | 6 | 28.75 | 25.00 | 12 | 5 | 12 |
| L66 | 2 | 41 | 6.5 | 6 | 5.28 | 6.70 | 0.68 | 0.19 | 748.99 | 4.56 | 7.93 | 4.50 | 15 | 38.75 | 46.25 | 18 | 13 | 6 |
| L67 | 2 | 49 | 6.2 | 9 | 12.17 | 9.40 | 0.87 | 0.21 | 711.99 | 2.68 | 7.20 | 4.50 | 6 | 31.25 | 25.00 | 19 | 11 | 3 |
| L68 | 1 | 48 | 5 | 6 | 1.56 | 7.60 | 0.75 | 0.13 | 678.00 | 3.35 | 6.43 | 5.00 | 2 | 26.25 | 25.00 | 7 | 11 | 15 |
| L69 | 1 | 26 | 7.4 | 9 | 10.29 | 9.50 | 1.15 | 0.33 | 789.99 | 3.16 | 7.48 | 4.50 | 9 | 43.75 | 37.50 | 33 | 10 | 5 |
| L70 | 2 | 50 | 8.8 | 9 | 8.83 | 13.80 | 1.11 | 0.25 | 1016.01 | 4.51 | 6.80 | 3.10 | 10 | 33.75 | 27.50 | 5 | 10 | 19 |
| L71 | 1 | 43 | 4.8 | 10 | 6.91 | 13.40 | 1.26 | 0.22 | 639.99 | 3.15 | 8.66 | 4.50 | 10 | 43.75 | 48.75 | 21 | 3 | 10 |
| L72 | 1 | 38 | 8.8 | 16 | 115.35 | 11.60 | 0.96 | 0.20 | 1173.00 | 4.84 | 6.64 | 4.70 | 3 | 33.75 | 31.25 | 19 | 19 | 11 |
| L73 | 1 | 40 | 6.4 | 10 | 15.10 | 9.20 | 0.82 | 0.16 | 690.00 | 4.87 | 6.33 | 4.50 | 8 | 46.25 | 40.00 | 25 | 5 | 4 |
| L74 | 2 | 51 | 4.2 | 10 | 12.73 | 6.30 | 0.99 | 0.18 | 663.99 | 2.96 | 6.00 | 5.20 | 5 | 40.00 | 33.75 | 20 | 10 | 2 |
| L75 | 1 | 40 | 2.5 | 10 | 19.99 | 9.70 | 0.86 | 0.37 | 795.99 | 4.12 | 5.00 | 7.90 | 4 | 33.75 | 27.50 | 23 | 15 | 6 |
| L76 | 2 | 65 | 4.9 | 6 | 3.25 | 10.30 | 0.80 | 0.23 | 726.99 | 2.74 | 6.26 | 4.50 | 7 | 36.25 | 30.00 | 4 | 7 | 9 |
| L77 | 1 | 49 | 7.2 | 17 | 110.56 | 16.70 | 1.16 | 0.31 | 799.00 | 18.01 | 4.48 | 5.00 | 6 | 31.25 | 25.00 | 43 | 14 | 14 |
| L78 | 2 | 29 | 8.2 | 13 | 43.91 | 11.90 | 0.83 | 0.16 | 1248.00 | 6.16 | 6.83 | 3.70 | 7 | 41.25 | 35.00 | 27 | 17 | 8 |
| L79 | 1 | 54 | 4.8 | 9 | 2.48 | 6.50 | 0.98 | 0.30 | 662.00 | 5.43 | 3.91 | 6.50 | 0 | 25.00 | 25.00 | 9 | 9 | 19 |
| L80 | 2 | 31 | 6.8 | 9 | 6.24 | 8.80 | 0.68 | 0.14 | 758.01 | 6.32 | 6.33 | 4.50 | 14 | 68.75 | 62.50 | 6 | 21 | 11 |
| L81 | 1 | 45 | 5.4 | 7 | 8.71 | 10.10 | 1.24 | 0.29 | 737.00 | 4.64 | 6.78 | 4.50 | 9 | 40.00 | 33.75 | 18 | 9 | 9 |
| L82 | 1 | 35 | 6 | 12 | 33.87 | 6.70 | 0.48 | 0.24 | 803.99 | 4.24 | 7.33 | 4.50 | 13 | 62.50 | 56.25 | 28 | 15 | 4 |
| L83 | 1 | 33 | 6.3 | 7 | 2.38 | 9.10 | 0.96 | 0.25 | 724.01 | 4.82 | 4.34 | 5.00 | 5 | 31.25 | 25.00 | 20 | 6 | 15 |
| L84 | 2 | 50 | 9.1 | 12 | 25.88 | 9.30 | 0.84 | 0.24 | 1148.01 | 2.74 | 7.13 | 4.50 | 7 | 36.25 | 27.50 | 12 | 6 | 6 |
| L85 | 1 | 36 | 5.4 | 7 | 3.87 | 8.80 | 0.72 | 0.17 | 803.01 | 4.70 | 5.83 | 4.70 | 0 | 25.00 | 25.00 | 7 | 16 | 12 |
| L86 | 1 | 35 | 12.4 | 15 | 109.25 | 16.10 | 0.81 | 0.15 | 1395.00 | 3.89 | 7.53 | 5.00 | 5 | 47.50 | 41.25 | 13 | 6 | 5 |
| L87 | 2 | 37 | 9.3 | 8 | 5.48 | 9.70 | 0.91 | 0.37 | 1115.01 | 4.13 | 8.59 | 6.60 | 11 | 36.25 | 46.25 | 12 | 9 | 9 |
| L88 | 1 | 29 | 17.6 | 15 | 76.03 | 11.30 | 1.42 | 0.30 | 1446.99 | 13.22 | 6.16 | 4.10 | 3 | 37.50 | 31.25 | 19 | 7 | 7 |
| L89 | 2 | 24 | 9.3 | 8 | 5.06 | 7.80 | 1.09 | 0.22 | 1167.00 | 4.79 | 7.04 | 4.50 | 8 | 36.25 | 30.00 | 16 | 3 | 12 |
| L90 | 2 | 30 | 10.7 | 10 | 13.89 | 8.80 | 0.73 | 0.17 | 1184.01 | 2.67 | 5.66 | 3.90 | 4 | 33.75 | 27.50 | 29 | 9 | 7 |
| L91 | 1 | 51 | 7.1 | 9 | 4.71 | 9.10 | 0.79 | 0.13 | 717.00 | 4.73 | 7.48 | 4.50 | 5 | 31.25 | 25.00 | 0 | 17 | 12 |
| L92 | 1 | 57 | 7.7 | 8 | 3.17 | 11.10 | 1.08 | 0.18 | 889.01 | 2.62 | 6.50 | 3.40 | 7 | 62.50 | 56.25 | 0 | 12 | 5 |
| L93 | 2 | 46 | 7.4 | 8 | 3.35 | 5.90 | 0.57 | 0.15 | 853.00 | 8.21 | 7.86 | 4.50 | 4 | 31.25 | 25.00 | 9 | 10 | 10 |
| L94 | 1 | 53 | 8.8 | 14 | 117.80 | 11.80 | 0.91 | 0.23 | 1068.00 | 8.32 | 3.65 | 4.30 | 6 | 32.50 | 26.25 | 21 | 10 | 16 |
| L95 | 1 | 32 | 10.6 | 7 | 5.91 | 8.60 | 0.91 | 0.23 | 1236.01 | 4.73 | 8.28 | 5.00 | 9 | 58.75 | 52.50 | 17 | 15 | 21 |
| L96 | 1 | 46 | 13.4 | 9 | 7.77 | 3.86 | 0.84 | 0.34 | 1213.01 | 3.75 | 3.41 | 5.00 | 12 | 40.00 | 33.75 | 18 | 8 | 3 |
| L97 | 2 | 50 | 7.3 | 9 | 10.57 | 7.50 | 0.74 | 0.18 | 841.00 | 3.82 | 7.68 | 2.90 | 7 | 31.25 | 25.00 | 7 | 7 | 15 |
| L98 | 1 | 39 | 8.3 | 16 | 143.69 | 10.80 | 0.87 | 0.26 | 983.01 | 12.54 | 6.16 | 6.60 | 7 | 52.50 | 53.75 | 27 | 13 | 18 |
| L99 | 1 | 31 | 14.1 | 10 | 3.97 | 6.90 | 0.74 | 0.16 | 1059.99 | 3.78 | 9.11 | 5.00 | 2 | 31.25 | 25.00 | 21 | 16 | 4 |
| L100 | 1 | 44 | 6.3 | 8 | 2.32 | 8.90 | 0.61 | 0.21 | 759.00 | 4.15 | 6.66 | 3.50 | 7 | 31.25 | 25.00 | 16 | 15 | 7 |

PASI, psoriasis area and severity index; BSA, body surface area; SCC-Ag, squamous cell carcinoma antigen; TNF-α, tumor necrosis factor–α; C3, complement 3; C4, complement 4; IL, interleukin; DLQI, Dermatology Life Quality Index; SAS, Self-Rating Anxiety Scale; SDS, Self-Rating Depression Scale; XQ, Xerostomia Questionnaire; CCS, Cleveland Clinic Score; PSQI, Pittsburgh Sleep Quality Index.

^a^1, male; 2, female.

# Supplementary Table S3 Introduction of the experts

| Experts | Professional position | Education background | Experience(years) |
| --- | --- | --- | --- |
| Expert1 | Student | Master | 4 |
| Expert2 | Student | Master | 6 |
| Expert3 | Doctor-in-charge | Master | 12 |
| Expert4 | Doctor-in-charge | Master | 12 |
| Expert5 | Doctor-in-charge | PhD | 17 |
| Expert6 | Associate Professor | Master | 24 |
| Expert7 | Associate Professor | PhD | 27 |
| Expert8 | Professor | PhD | 31 |
| Expert9 | Professor | PhD | 37 |
| Expert10 | Professor | PhD | 42 |

# Supplementary Table S4 Expert scores of the indexes

| Indexes | Expert1 | Expert2 | Expert3 | Expert4 | Expert5 | Expert6 | Expert7 | Expert8 | Expert9 | Expert10 |
| --- | --- | --- | --- | --- | --- | --- | --- | --- | --- | --- |
| PASI | 10 | 10 | 10 | 8 | 10 | 8 | 10 | 9 | 10 | 9 |
| BSA | 6 | 10 | 7 | 9 | 9 | 8 | 6 | 7 | 8 | 7 |
| C3 | 2 | 1 | 3 | 2 | 3 | 4 | 3 | 3 | 5 | 5 |
| C4 | 3 | 2 | 3 | 5 | 4 | 4 | 3 | 4 | 3 | 5 |
| XQ | 4 | 7 | 5 | 5 | 4 | 5 | 7 | 6 | 3 | 2 |
| PSQI | 5 | 6 | 6 | 6 | 5 | 4 | 6 | 6 | 5 | 5 |
| CCS | 5 | 6 | 5 | 5 | 3 | 3 | 3 | 5 | 5 | 4 |
| DLQI | 5 | 5 | 5 | 3 | 3 | 5 | 4 | 6 | 5 | 3 |
| SDS | 5 | 6 | 4 | 5 | 6 | 4 | 4 | 5 | 3 | 5 |
| SAS | 5 | 5 | 4 | 4 | 6 | 3 | 4 | 5 | 4 | 5 |
| IL-10 | 4 | 4 | 3 | 5 | 3 | 2 | 3 | 4 | 3 | 4 |
| IL-22 | 7 | 6 | 5 | 5 | 7 | 6 | 6 | 7 | 6 | 7 |
| IL-17 | 9 | 7 | 9 | 8 | 9 | 9 | 8 | 9 | 7 | 8 |
| IL-23 | 7 | 6 | 7 | 7 | 7 | 8 | 7 | 7 | 7 | 8 |
| Skin lesion conditions | 9 | 9 | 9 | 9 | 8 | 9 | 8 | 9 | 9 | 9 |
| Laboratory indexes | 7 | 5 | 6 | 6 | 5 | 6 | 5 | 5 | 4 | 6 |
| Quality of life | 5 | 4 | 6 | 5 | 4 | 5 | 4 | 4 | 4 | 7 |
| Accompanying symptoms | 5 | 4 | 5 | 6 | 4 | 4 | 3 | 4 | 3 | 5 |
| TNF-α | 3 | 3 | 5 | 6 | 4 | 5 | 3 | 3 | 4 | 5 |
| SCC-Ag | 2 | 5 | 4 | 6 | 4 | 5 | 4 | 5 | 5 | 5 |
| Complement | 1 | 3 | 2 | 3 | 1 | 4 | 1 | 2 | 3 | 1 |
| Interleukin | 8 | 9 | 9 | 8 | 7 | 8 | 7 | 9 | 8 | 7 |

# Supplementary Table S5 Results of AHP and the consistency test

| Indexes | Weights | CI | CR |
| --- | --- | --- | --- |
| PASI | 0.6296 | -0.0005 | 0.0006 |
| BSA | 0.3704 | -0.0005 | 0.0006 |
| C3 | 0.6667 | -0.0004 | 0.0005 |
| C4 | 0.3333 | -0.0004 | 0.0005 |
| SCC | 0.1474 | 0.0812 | 0.0893 |
| Complement | 0.067 | 0.0812 | 0.0893 |
| Interleukin | 0.5291 | 0.0812 | 0.0893 |
| TNF-α | 0.2565 | 0.0812 | 0.0893 |
| IL-10 | 0.0955 | 0.0361 | 0.0397 |
| IL-17 | 0.3435 | 0.0361 | 0.0397 |
| IL-22 | 0.2909 | 0.0361 | 0.0397 |
| IL-23 | 0.2702 | 0.0361 | 0.0397 |
| DLQI | 0.3500 | 0.0276 | 0.0576 |
| SAS | 0.2517 | 0.0276 | 0.0576 |
| SDS | 0.3982 | 0.0276 | 0.0576 |
| XQ | 0.3833 | 0.0241 | 0.0502 |
| CCS | 0.1767 | 0.0241 | 0.0502 |
| PSQI | 0.4399 | 0.0241 | 0.0502 |
| Skin lesion conditions | 0.4966 | 0.0026 | 0.0029 |
| Laboratory indexes | 0.2658 | 0.0026 | 0.0029 |
| Quality of Life | 0.0829 | 0.0026 | 0.0029 |
| Accompanying symptoms | 0.1548 | 0.0026 | 0.0029 |

CI, consensus index; CR, consistency ratio.

# Supplementary Table S6 The SD of 100 patients

| Patients | PASI | BSA | SCC | TNF | C3 | C4 | IL23 | IL22 | IL17 | IL10 | DLQI | SAS | SDS | XQ | CCS | PSQI |
| --- | --- | --- | --- | --- | --- | --- | --- | --- | --- | --- | --- | --- | --- | --- | --- | --- |
| L1 | 0.3478 | 0.3122 | 0.6065 | 0.7716 | 0.4871 | 0.9894 | 0.3177 | 0.6654 | 0.9271 | 0.4494 | 0.5833 | 0.8344 | 1.0000 | 0.3672 | 0.4119 | 0.8639 |
| L2 | 0.3901 | 0.5289 | 0.8802 | 0.8432 | 0.5445 | 0.9900 | 0.5471 | 0.8866 | 0.8247 | 0.3867 | 0.4633 | 0.5855 | 0.7627 | 0.5242 | 0.3333 | 0.2925 |
| L3 | 0.6037 | 0.7527 | 0.9525 | 0.8101 | 0.5932 | 0.9937 | 0.7132 | 0.9966 | 0.5163 | 0.4106 | 0.5833 | 0.5855 | 0.7627 | 0.2829 | 0.5500 | 0.2772 |
| L4 | 0.4923 | 0.5352 | 0.9602 | 0.8703 | 0.5379 | 0.9888 | 0.5096 | 0.9709 | 0.8133 | 0.4494 | 0.3700 | 0.7444 | 0.9161 | 0.5911 | 0.2929 | 0.4949 |
| L5 | 0.6037 | 0.7527 | 0.9865 | 0.4912 | 0.5719 | 0.9950 | 0.7313 | 0.4118 | 0.9504 | 0.2618 | 0.3700 | 0.8344 | 1.0000 | 0.6398 | 0.3833 | 1.0000 |
| L6 | 0.4678 | 0.5289 | 0.9719 | 0.8166 | 0.6004 | 0.9900 | 0.4040 | 0.8567 | 0.6611 | 0.2899 | 0.9033 | 0.8344 | 1.0000 | 1.0000 | 0.2405 | 0.5374 |
| L7 | 0.2621 | 0.2875 | 0.5323 | 0.7106 | 0.6454 | 0.9981 | 0.4145 | 0.4150 | 0.7610 | 0.3867 | 0.6533 | 0.6883 | 0.8628 | 0.5242 | 0.2762 | 0.2925 |
| L8 | 0.6098 | 0.6511 | 0.9546 | 0.8635 | 0.5860 | 0.9727 | 0.8882 | 0.7906 | 0.9851 | 0.4361 | 0.6533 | 0.8983 | 0.9161 | 1.0000 | 0.4762 | 0.2653 |
| L9 | 0.4100 | 0.4650 | 0.8717 | 0.8036 | 0.2500 | 0.9813 | 0.3507 | 0.9709 | 0.4628 | 0.6753 | 0.5833 | 0.7160 | 0.8892 | 0.4122 | 0.2500 | 0.3878 |
| L10 | 0.4537 | 0.6150 | 0.9848 | 0.8166 | 0.7176 | 0.9962 | 0.3590 | 0.9451 | 0.3154 | 0.4494 | 0.5833 | 0.5387 | 0.7158 | 1.0000 | 0.2500 | 0.7415 |
| L11 | 0.4774 | 0.2584 | 0.3200 | 0.7716 | 0.7344 | 0.9962 | 0.3468 | 0.9065 | 0.9007 | 0.4494 | 0.3700 | 0.6100 | 0.6495 | 0.3295 | 0.2286 | 0.6854 |
| L12 | 0.6098 | 0.6888 | 0.9996 | 0.8432 | 0.4994 | 0.9975 | 0.7453 | 0.9517 | 0.7230 | 0.4494 | 0.7300 | 1.0000 | 1.0000 | 0.5911 | 0.2500 | 0.8010 |
| L13 | 0.5236 | 0.4285 | 0.9406 | 0.8911 | 0.6226 | 0.9937 | 0.5361 | 0.9166 | 1.0000 | 0.3537 | 0.3333 | 0.3373 | 0.2500 | 1.0000 | 0.2762 | 0.3333 |
| L14 | 0.3940 | 0.3777 | 0.7786 | 0.9051 | 0.6078 | 0.9919 | 0.4653 | 0.9262 | 0.7350 | 0.6943 | 0.5833 | 0.8344 | 1.0000 | 0.5680 | 0.2762 | 0.4201 |
| L15 | 0.5399 | 0.4596 | 0.9688 | 0.7843 | 0.7010 | 0.9919 | 0.5997 | 0.7382 | 0.9486 | 0.4494 | 0.8133 | 0.8344 | 1.0000 | 0.5242 | 0.2262 | 0.5374 |
| L16 | 0.7148 | 0.7123 | 0.9925 | 0.8499 | 0.4751 | 0.9881 | 0.7918 | 0.9364 | 0.5163 | 0.3241 | 0.5200 | 0.8344 | 1.0000 | 0.5457 | 0.2762 | 0.6854 |
| L17 | 0.7079 | 0.6966 | 0.9590 | 0.8807 | 0.6609 | 0.9969 | 0.7772 | 0.9709 | 0.7502 | 0.4494 | 1.0000 | 1.0000 | 0.8628 | 0.7469 | 0.4119 | 0.5833 |
| L18 | 0.5289 | 0.3497 | 0.6582 | 0.8465 | 0.6929 | 0.9931 | 0.5584 | 0.7848 | 0.7610 | 0.3867 | 0.4633 | 0.8344 | 1.0000 | 0.7469 | 0.2929 | 0.9303 |
| L19 | 0.6476 | 0.5107 | 0.8175 | 0.8807 | 0.6531 | 0.9944 | 0.7101 | 0.9473 | 0.8133 | 0.3867 | 0.5833 | 0.8344 | 1.0000 | 0.6151 | 0.2762 | 0.3588 |
| L20 | 0.4268 | 0.2544 | 0.2880 | 0.7047 | 0.5581 | 0.9925 | 0.3969 | 0.7673 | 0.4151 | 0.4106 | 0.8133 | 0.8983 | 1.0000 | 0.4122 | 0.2929 | 0.8010 |
| L21 | 0.5236 | 0.4542 | 0.8424 | 0.8166 | 0.5789 | 0.9950 | 0.5310 | 0.8222 | 0.6894 | 0.3867 | 0.5200 | 0.6883 | 0.8628 | 0.7757 | 0.4429 | 0.2653 |
| L22 | 0.5025 | 0.5414 | 0.9610 | 0.9775 | 0.8793 | 0.9956 | 0.5243 | 0.9113 | 0.6034 | 0.3867 | 0.4633 | 0.8036 | 0.9715 | 0.7189 | 0.2405 | 0.6854 |
| L23 | 0.5182 | 0.4437 | 0.7450 | 0.8841 | 0.5581 | 0.9969 | 0.5219 | 0.9337 | 0.7972 | 0.4494 | 0.6533 | 0.7444 | 0.9161 | 0.3413 | 0.3119 | 0.4558 |
| L24 | 0.5236 | 0.3735 | 0.7505 | 0.8669 | 0.6531 | 0.9956 | 0.5463 | 0.9065 | 0.6980 | 0.6206 | 0.5833 | 0.6353 | 0.8117 | 0.3672 | 0.2405 | 0.6327 |
| L25 | 0.4268 | 0.3535 | 0.6736 | 0.7165 | 0.6609 | 0.9981 | 0.3458 | 0.8366 | 0.7471 | 0.3867 | 0.5200 | 0.6883 | 0.8628 | 0.4289 | 0.2762 | 0.3333 |
| L26 | 0.4630 | 0.4873 | 0.8241 | 0.8365 | 0.6768 | 0.9944 | 0.4976 | 0.7696 | 0.6405 | 0.4494 | 0.6533 | 0.7160 | 0.8892 | 0.5457 | 0.4119 | 0.4949 |
| L27 | 0.7650 | 0.4705 | 0.9748 | 0.8911 | 0.7429 | 0.9956 | 0.6914 | 0.8939 | 0.7411 | 0.4494 | 0.7300 | 0.8344 | 1.0000 | 0.5242 | 0.2286 | 0.8639 |
| L28 | 0.4774 | 0.3216 | 0.6516 | 0.6129 | 0.6609 | 0.9969 | 0.3969 | 0.7598 | 0.5957 | 0.4494 | 0.4133 | 0.4744 | 0.6495 | 0.6917 | 0.3119 | 0.8010 |
| L29 | 0.3007 | 0.4138 | 0.7449 | 0.7654 | 0.4520 | 0.9850 | 0.3856 | 0.7734 | 0.7844 | 0.4494 | 0.5833 | 0.7444 | 0.9161 | 0.5680 | 0.2762 | 0.5833 |
| L30 | 0.5344 | 0.4816 | 0.8414 | 0.8068 | 0.9184 | 0.9975 | 0.5770 | 0.9626 | 0.5405 | 0.4494 | 0.6533 | 0.9653 | 1.0000 | 0.6917 | 0.2500 | 0.5374 |
| L31 | 0.5916 | 0.4650 | 0.9681 | 0.8669 | 0.6688 | 0.9956 | 0.6711 | 0.9709 | 0.7305 | 0.4494 | 1.0000 | 1.0000 | 1.0000 | 0.6151 | 0.2762 | 0.5374 |
| L32 | 0.3510 | 0.3998 | 0.4732 | 0.7907 | 0.6151 | 0.2500 | 0.4378 | 0.4499 | 0.6937 | 0.3867 | 0.5200 | 0.6883 | 0.8628 | 0.2906 | 0.3571 | 0.6327 |
| L33 | 0.8892 | 0.8209 | 0.9410 | 0.8981 | 0.5247 | 0.9925 | 0.8712 | 0.9506 | 0.6405 | 0.4494 | 0.5200 | 0.7444 | 0.7390 | 0.4836 | 0.2762 | 0.2500 |
| L34 | 0.7577 | 0.3953 | 0.7825 | 0.7971 | 0.7429 | 1.0000 | 0.7907 | 0.9359 | 0.5818 | 0.4494 | 0.6533 | 0.7160 | 0.8892 | 0.7189 | 0.4429 | 0.4558 |
| L35 | 0.6160 | 0.6364 | 0.9712 | 0.8068 | 0.7602 | 0.9894 | 0.7442 | 0.8346 | 0.7350 | 0.4494 | 0.3700 | 0.7736 | 0.9435 | 0.4836 | 0.2500 | 0.8010 |
| L36 | 0.5977 | 1.0000 | 1.0000 | 0.9122 | 0.6151 | 0.9931 | 0.7164 | 0.9709 | 0.5346 | 0.4494 | 0.8133 | 0.8983 | 1.0000 | 0.8053 | 0.2762 | 0.8639 |
| L37 | 0.4400 | 0.5047 | 0.9310 | 0.9887 | 0.2757 | 0.9950 | 0.6174 | 0.9621 | 0.8732 | 0.4494 | 1.0000 | 0.8036 | 0.9715 | 0.3964 | 0.3571 | 0.2925 |
| L38 | 0.7289 | 0.5167 | 0.8966 | 0.7875 | 0.3801 | 0.9863 | 0.8907 | 0.9709 | 0.7230 | 0.4494 | 0.5833 | 0.8660 | 1.0000 | 0.5457 | 0.2405 | 0.7415 |
| L39 | 0.6806 | 0.4650 | 0.8393 | 0.9016 | 0.7093 | 1.0000 | 0.7639 | 0.9709 | 0.6178 | 0.4494 | 0.8133 | 0.8983 | 1.0000 | 0.3538 | 0.3119 | 0.6327 |
| L40 | 0.4100 | 0.5542 | 0.9506 | 0.9775 | 0.7093 | 0.9956 | 0.5056 | 0.9709 | 0.7112 | 0.4494 | 0.6533 | 0.6353 | 0.8117 | 1.0000 | 0.2333 | 0.2500 |
| L41 | 0.7360 | 0.6010 | 0.9464 | 0.8669 | 0.6768 | 0.9956 | 0.7749 | 0.8371 | 0.5163 | 0.4232 | 0.3700 | 0.5617 | 0.7390 | 0.4836 | 0.2333 | 0.5833 |
| L42 | 0.4141 | 0.2828 | 0.4754 | 0.7971 | 0.9792 | 0.9919 | 0.3838 | 0.4670 | 0.6021 | 0.4494 | 0.3333 | 0.3829 | 0.5496 | 0.4836 | 0.2500 | 0.3112 |
| L43 | 0.7218 | 0.5873 | 0.9522 | 0.9372 | 0.6848 | 0.9937 | 0.8169 | 0.9709 | 0.2814 | 0.4494 | 0.3033 | 0.4545 | 0.4793 | 0.3295 | 0.4762 | 0.3112 |
| L44 | 0.5567 | 0.3574 | 0.6245 | 0.2500 | 0.6226 | 0.9975 | 0.5318 | 0.7810 | 0.6473 | 0.3867 | 1.0000 | 0.8344 | 1.0000 | 0.2760 | 0.3119 | 0.7415 |
| L45 | 1.0000 | 0.9603 | 0.9925 | 0.8635 | 0.7260 | 0.9956 | 1.0000 | 0.9262 | 0.7487 | 0.3434 | 0.5833 | 0.8036 | 0.9715 | 1.0000 | 0.3833 | 0.6854 |
| L46 | 0.4726 | 0.4596 | 0.8505 | 0.8807 | 0.7010 | 0.9969 | 0.4072 | 0.9294 | 0.7200 | 0.3867 | 0.5200 | 0.7736 | 0.9435 | 0.6398 | 0.2929 | 0.3112 |
| L47 | 0.4923 | 0.5607 | 0.9427 | 1.0000 | 0.5650 | 0.9931 | 0.4921 | 0.9566 | 0.7350 | 0.4361 | 0.5833 | 0.8036 | 0.9715 | 0.3084 | 0.3571 | 0.8010 |
| L48 | 0.8649 | 0.8209 | 0.9566 | 0.8398 | 0.6226 | 0.9956 | 0.7839 | 0.8608 | 0.5548 | 0.3241 | 0.8133 | 0.9653 | 1.0000 | 0.6151 | 0.2333 | 0.2517 |
| L49 | 0.9478 | 0.6660 | 0.9180 | 0.9336 | 0.5119 | 0.9900 | 0.8724 | 0.9489 | 0.5174 | 0.3867 | 0.5200 | 0.8344 | 1.0000 | 0.8990 | 0.3119 | 0.2925 |
| L50 | 0.3863 | 0.4091 | 0.7986 | 0.7225 | 0.6848 | 0.9937 | 0.4040 | 0.8217 | 0.6625 | 0.3867 | 0.5833 | 0.5855 | 0.7627 | 0.4122 | 0.2405 | 0.2653 |
| L51 | 0.5977 | 0.6221 | 0.8305 | 0.8807 | 0.7260 | 0.9975 | 0.5610 | 0.8237 | 0.4679 | 0.3867 | 0.5833 | 0.6614 | 0.8370 | 0.3672 | 0.2619 | 0.4558 |
| L52 | 0.2732 | 0.2900 | 0.5630 | 0.5407 | 0.6377 | 0.9937 | 0.2655 | 0.2500 | 0.6865 | 1.0000 | 0.5200 | 0.7160 | 0.8892 | 0.6917 | 0.5119 | 0.3588 |
| L53 | 0.6222 | 0.5873 | 0.9628 | 0.5820 | 0.5119 | 0.9925 | 0.7238 | 0.8486 | 0.4558 | 0.3867 | 0.8133 | 1.0000 | 1.0000 | 0.5680 | 0.2500 | 0.5833 |
| L54 | 0.4923 | 0.4186 | 0.7972 | 0.7560 | 0.5581 | 0.9956 | 0.4638 | 0.8502 | 0.4783 | 0.3867 | 0.4633 | 0.5617 | 0.7390 | 0.5911 | 0.2762 | 0.3333 |
| L55 | 0.5798 | 0.5107 | 0.8954 | 0.8365 | 0.7093 | 0.9987 | 0.7873 | 0.9065 | 0.3794 | 0.7137 | 0.4133 | 0.7160 | 0.8892 | 0.3084 | 0.4762 | 0.7415 |
| L56 | 0.6541 | 0.5107 | 0.8943 | 0.7907 | 0.4932 | 0.9888 | 0.7628 | 0.8639 | 0.5893 | 0.4915 | 0.9033 | 1.0000 | 1.0000 | 0.4289 | 0.2619 | 0.2568 |
| L57 | 0.5236 | 0.6364 | 0.9798 | 0.8004 | 0.5719 | 0.9900 | 0.5707 | 0.8552 | 0.4151 | 0.2500 | 0.5200 | 0.8344 | 1.0000 | 0.6654 | 0.3119 | 0.5833 |
| L58 | 0.5681 | 0.5289 | 0.9172 | 0.8004 | 0.4871 | 0.9937 | 0.7007 | 0.9177 | 0.4731 | 0.3867 | 0.6533 | 0.6614 | 0.8370 | 0.3672 | 0.2500 | 1.0000 |
| L59 | 0.7360 | 0.6010 | 0.9726 | 0.8738 | 0.7602 | 0.9610 | 0.5997 | 0.9359 | 0.3653 | 0.3241 | 0.5833 | 0.4545 | 0.6285 | 1.0000 | 0.2762 | 0.6854 |
| L60 | 0.5129 | 0.6080 | 0.9686 | 0.9628 | 0.6688 | 0.9956 | 0.5394 | 0.8829 | 0.4430 | 0.4361 | 0.6533 | 0.6614 | 0.8370 | 0.4646 | 0.3333 | 0.7415 |
| L61 | 0.5289 | 0.5352 | 0.9775 | 0.8332 | 0.4464 | 0.9906 | 0.6193 | 0.5988 | 0.3492 | 0.2823 | 1.0000 | 1.0000 | 1.0000 | 0.5680 | 0.3571 | 0.8010 |
| L62 | 0.3478 | 0.3613 | 0.7253 | 0.8432 | 0.6454 | 0.9956 | 0.3832 | 0.5301 | 0.4588 | 0.4494 | 1.0000 | 0.8660 | 1.0000 | 0.7189 | 0.3571 | 0.8639 |
| L63 | 0.5567 | 0.5352 | 0.9178 | 0.6645 | 0.6078 | 0.9931 | 0.5979 | 0.8291 | 0.6625 | 0.3753 | 0.3700 | 0.5855 | 0.7627 | 0.5457 | 0.2286 | 0.2653 |
| L64 | 0.3210 | 0.4989 | 0.9478 | 0.9925 | 0.5119 | 0.9975 | 0.4442 | 0.8736 | 0.4430 | 0.4771 | 0.5200 | 0.3236 | 0.4793 | 0.7469 | 0.3333 | 0.8010 |
| L65 | 0.4774 | 0.4760 | 0.6628 | 0.7498 | 0.5513 | 0.9894 | 0.3507 | 0.8639 | 0.9814 | 0.3867 | 0.5200 | 0.8983 | 1.0000 | 0.6398 | 0.3833 | 0.4201 |
| L66 | 0.6098 | 0.7123 | 0.9575 | 0.8911 | 0.8046 | 0.9962 | 0.7164 | 0.8944 | 0.2921 | 0.3643 | 0.2500 | 0.6614 | 0.5880 | 0.5035 | 0.2405 | 0.3878 |
| L67 | 0.6285 | 0.5047 | 0.8891 | 0.8004 | 0.6454 | 0.9950 | 0.7562 | 0.9966 | 0.3341 | 0.7137 | 0.5200 | 0.8344 | 1.0000 | 0.4836 | 0.2619 | 0.6327 |
| L68 | 0.7079 | 0.6966 | 0.9959 | 0.8601 | 0.7429 | 1.0000 | 0.7941 | 0.9593 | 0.3917 | 0.2751 | 0.8133 | 0.9653 | 1.0000 | 0.7757 | 0.2619 | 0.8010 |
| L69 | 0.5567 | 0.5228 | 0.9075 | 0.7971 | 0.4577 | 0.9875 | 0.6741 | 0.9698 | 0.3165 | 0.3985 | 0.3700 | 0.5617 | 0.7390 | 0.2906 | 0.2762 | 0.3112 |
| L70 | 0.4824 | 0.5047 | 0.9219 | 0.6673 | 0.4811 | 0.9925 | 0.4743 | 0.8970 | 0.3623 | 0.5530 | 0.3333 | 0.7736 | 0.9435 | 0.8357 | 0.2762 | 0.6854 |
| L71 | 0.7218 | 0.4816 | 0.9410 | 0.6786 | 0.3992 | 0.9944 | 0.8379 | 0.9704 | 0.2623 | 0.3867 | 0.3333 | 0.5617 | 0.5496 | 0.4463 | 0.4429 | 0.2568 |
| L72 | 0.4824 | 0.2521 | 0.2806 | 0.7315 | 0.5789 | 0.9956 | 0.3688 | 0.8798 | 0.3746 | 1.0000 | 0.7300 | 0.7736 | 0.8628 | 0.4836 | 0.2333 | 0.4558 |
| L73 | 0.6160 | 0.4650 | 0.8611 | 0.8068 | 0.6848 | 0.9981 | 0.7805 | 0.8783 | 0.4001 | 0.3867 | 0.4133 | 0.5165 | 0.6932 | 0.3814 | 0.3833 | 0.4201 |
| L74 | 0.7650 | 0.4760 | 0.8837 | 0.9051 | 0.5581 | 0.9969 | 0.8100 | 0.9809 | 0.4297 | 0.2899 | 0.5833 | 0.6353 | 0.8117 | 0.4646 | 0.2762 | 0.7415 |
| L75 | 0.8974 | 0.4386 | 0.8157 | 0.7907 | 0.6531 | 0.9850 | 0.6681 | 0.9177 | 0.5346 | 0.3867 | 0.6533 | 0.7736 | 0.9435 | 0.4122 | 0.2286 | 0.8639 |
| L76 | 0.7148 | 0.7203 | 0.9784 | 0.7716 | 0.7010 | 0.9937 | 0.7399 | 0.9932 | 0.4062 | 0.4494 | 0.4633 | 0.7160 | 0.8892 | 0.8670 | 0.3333 | 0.6327 |
| L77 | 0.5681 | 0.2500 | 0.2916 | 0.5896 | 0.4520 | 0.9888 | 0.6651 | 0.3820 | 0.5983 | 0.6385 | 0.5200 | 0.8344 | 1.0000 | 0.2500 | 0.2333 | 0.4949 |
| L78 | 0.5129 | 0.3184 | 0.6189 | 0.7225 | 0.6768 | 0.9981 | 0.3280 | 0.8133 | 0.3601 | 0.3867 | 0.4633 | 0.6100 | 0.7869 | 0.3538 | 0.2262 | 0.3333 |
| L79 | 0.7218 | 0.5107 | 0.9863 | 0.8981 | 0.5650 | 0.9894 | 0.8123 | 0.8497 | 0.6751 | 0.3867 | 1.0000 | 1.0000 | 1.0000 | 0.7189 | 0.2929 | 0.5374 |
| L80 | 0.5916 | 0.4930 | 0.9478 | 0.8199 | 0.8046 | 0.9994 | 0.7070 | 0.8055 | 0.4001 | 0.3867 | 0.2533 | 0.2500 | 0.3768 | 0.8053 | 0.2500 | 0.2568 |
| L81 | 0.6806 | 0.6437 | 0.9230 | 0.7780 | 0.4092 | 0.9900 | 0.7291 | 0.8902 | 0.3638 | 0.3867 | 0.3700 | 0.6353 | 0.8117 | 0.5035 | 0.2929 | 0.4201 |
| L82 | 0.6412 | 0.3574 | 0.6964 | 0.8911 | 1.0000 | 0.9931 | 0.6601 | 0.9113 | 0.3257 | 0.3867 | 0.2633 | 0.2987 | 0.4474 | 0.3413 | 0.2286 | 0.4949 |
| L83 | 0.6222 | 0.6511 | 0.9874 | 0.8101 | 0.5789 | 0.9925 | 0.7431 | 0.8809 | 0.6164 | 0.2899 | 0.5833 | 0.8344 | 1.0000 | 0.4646 | 0.3571 | 0.7415 |
| L84 | 0.4678 | 0.3777 | 0.7634 | 0.8036 | 0.6688 | 0.9931 | 0.3838 | 0.9932 | 0.3388 | 0.3867 | 0.4633 | 0.7160 | 0.9435 | 0.6398 | 0.3571 | 0.3112 |
| L85 | 0.6806 | 0.6511 | 0.9720 | 0.8199 | 0.7689 | 0.9975 | 0.6611 | 0.8871 | 0.4459 | 0.3867 | 1.0000 | 1.0000 | 1.0000 | 0.7757 | 0.2262 | 0.6327 |
| L86 | 0.3384 | 0.2682 | 0.2949 | 0.6050 | 0.6929 | 0.9987 | 0.2662 | 0.9300 | 0.3136 | 0.4232 | 0.5833 | 0.4951 | 0.6711 | 0.6151 | 0.3571 | 0.3878 |
| L87 | 0.4583 | 0.6080 | 0.9555 | 0.7907 | 0.6151 | 0.9850 | 0.4046 | 0.9171 | 0.2646 | 0.3867 | 0.3033 | 0.7160 | 0.5880 | 0.6398 | 1.0000 | 0.6854 |
| L88 | 0.2500 | 0.2805 | 0.4206 | 0.7406 | 0.3296 | 0.9894 | 0.2500 | 0.5205 | 0.4151 | 0.3867 | 0.7300 | 0.6883 | 0.8628 | 0.4836 | 0.3333 | 0.4949 |
| L89 | 0.4583 | 0.5805 | 0.9598 | 0.8533 | 0.4932 | 0.9944 | 0.3724 | 0.8824 | 0.3449 | 0.3867 | 0.4133 | 0.7160 | 0.8892 | 0.5457 | 0.4429 | 0.5833 |
| L90 | 0.3979 | 0.4705 | 0.8726 | 0.8199 | 0.7602 | 0.9975 | 0.3624 | 0.9972 | 0.4628 | 0.3867 | 0.6533 | 0.7736 | 0.9435 | 0.3295 | 0.2929 | 0.5833 |
| L91 | 0.5739 | 0.5107 | 0.9634 | 0.8101 | 0.7093 | 1.0000 | 0.7507 | 0.8855 | 0.3165 | 0.3062 | 0.5833 | 0.8344 | 1.0000 | 1.0000 | 0.2262 | 0.3878 |
| L92 | 0.5399 | 0.6010 | 0.9792 | 0.7468 | 0.4994 | 0.9969 | 0.5796 | 1.0000 | 0.3859 | 0.2899 | 0.4633 | 0.2987 | 0.4474 | 1.0000 | 0.2500 | 0.6854 |
| L93 | 0.5567 | 0.6080 | 0.9773 | 0.9193 | 0.9085 | 0.9987 | 0.6127 | 0.7174 | 0.2956 | 0.2899 | 0.6533 | 0.8344 | 1.0000 | 0.7189 | 0.2762 | 0.4558 |
| L94 | 0.4824 | 0.2851 | 0.8929 | 0.7255 | 0.6151 | 0.9937 | 0.4364 | 0.7125 | 0.7127 | 0.3867 | 0.5200 | 0.8036 | 0.9715 | 0.4463 | 0.2762 | 0.2925 |
| L95 | 0.4019 | 0.6150 | 0.9511 | 0.8265 | 0.6151 | 0.9937 | 0.3341 | 0.8855 | 0.2763 | 0.3867 | 0.3700 | 0.3373 | 0.4961 | 0.5242 | 0.2286 | 0.2500 |
| L96 | 0.3104 | 0.4873 | 0.9324 | 0.9940 | 0.6688 | 0.9869 | 0.3463 | 0.9375 | 0.7487 | 0.4915 | 0.2800 | 0.6353 | 0.8117 | 0.5035 | 0.3119 | 0.8010 |
| L97 | 0.5624 | 0.5167 | 0.9047 | 0.8635 | 0.7515 | 0.9969 | 0.6241 | 0.9337 | 0.3051 | 0.6206 | 0.4633 | 0.8344 | 1.0000 | 0.7757 | 0.3333 | 0.3112 |
| L98 | 0.5077 | 0.2570 | 0.2500 | 0.7560 | 0.6454 | 0.9919 | 0.5000 | 0.5441 | 0.4151 | 0.7741 | 0.4633 | 0.4171 | 0.4793 | 0.3538 | 0.2405 | 0.2653 |
| L99 | 0.2939 | 0.4386 | 0.9709 | 0.8841 | 0.7515 | 0.9981 | 0.4420 | 0.9359 | 0.2500 | 0.5063 | 0.8133 | 0.8344 | 1.0000 | 0.4463 | 0.2262 | 0.7415 |
| L100 | 0.6222 | 0.6080 | 0.9880 | 0.8166 | 0.8697 | 0.9950 | 0.7059 | 0.9161 | 0.3730 | 0.2618 | 0.4633 | 0.8344 | 1.0000 | 0.5457 | 0.2286 | 0.5833 |
